# Traditional Uses, Chemical Constituents and Pharmacological Activities of the *Toona sinensis* Plant

**DOI:** 10.3390/molecules29030718

**Published:** 2024-02-04

**Authors:** Mengyao Zhao, Huiting Li, Rongshen Wang, Shuying Lan, Yuxin Wang, Yuhua Zhang, Haishan Sui, Wanzhong Li

**Affiliations:** 1 School of Pharmacy, Shandong Second Medical University, Weifang 261053, China; wfmc20221028@126.com (M.Z.); wnmclht@126.com (H.L.); wangrs199012@163.com (R.W.); 18878630861@163.com (S.L.); 18363412971@163.com (Y.W.); zhangyh@wfmc.edu.cn (Y.Z.); 2 Weifang City Inspection and Testing Center, Weifang 261100, China

**Keywords:** *Toona sinensis*, traditional uses, chemical constituent, pharmacological activity

## Abstract

*Toona sinensis* (A. Juss.) Roem., which is widely distributed in China, is a homologous plant resource of medicine and food. The leaves, seeds, barks, buds and pericarps of *T. sinensis* can be used as medicine with traditional efficacy. Due to its extensive use in traditional medicine in the ancient world, the *T. sinensis* plant has significant development potential. In this review, 206 compounds, including triterpenoids (**1**–**133**), sesquiterpenoids (**134**–**135**), diterpenoids (**136**–**142**), sterols (**143**–**147**), phenols (**148**–**167**), flavonoids (**168**–**186**), phenylpropanoids (**187**–**192**) and others (**193**–**206**), are isolated from the *T. sinensis* plant. The mass spectrum cracking laws of representative compounds (**64**, **128**, **129**, **154**–**156**, **175**, **177**, **179** and **183**) are reviewed, which are conducive to the discovery of novel active substances. Modern pharmacological studies have shown that *T. sinensis* extracts and their compounds have antidiabetic, antidiabetic nephropathy, antioxidant, anti-inflammatory, antitumor, hepatoprotective, antiviral, antibacterial, immunopotentiation and other biological activities. The traditional uses, chemical constituents, compound cracking laws and pharmacological activities of different parts of *T. sinensis* are reviewed, laying the foundation for improving the development and utilization of its medicinal value.

## 1. Introduction

The *Toona* genus (Meliaceae) comprises about 15 species, which are distributed from Asia to Oceania. Approximately four species, including *Toona sinensis* (A. Juss.) Roem, *Toona ciliata* M. Roem., *Toona microcarpa* (C. DC.) Harms and *Toona rubriflora* C. J. Tseng, are found in China with distribution in the south, southwest and north [1]. In addition to the characteristics of the species, *T. sinensis* seeds have membranous wings, which facilitate flying and spreading. More than 2000 years of cultivation history has resulted in the species’ strong cold resistance. Of course, it already had this genetic advantage, which made it widely cultivated on the lands of China [2,3]. *T. sinensis* has a long history of cultivation, wide distribution, strong adaptability and easy reproduction. It is a valuable multifunctional tree species that integrates food, medicine and materials, beautifies the environment and has significant potential for development and utilization [4,5].

**Figure 1:**
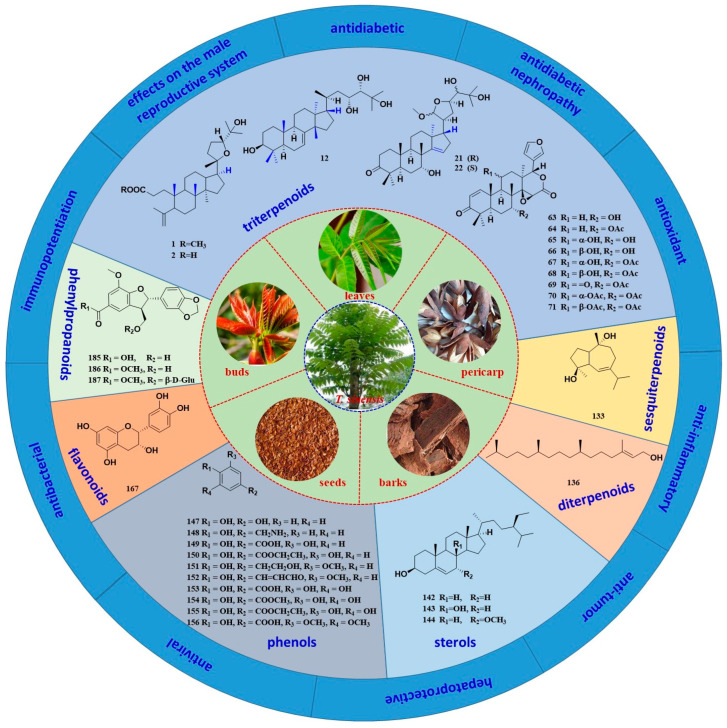
Different parts of the *T. sinensis* plant and its chemical constituents and pharmacological activities.

*T. sinensis* was first published in Tang Materia Medica, describing its efficacy in transforming food and medicine, which were widely used in traditional medicine in the ancient world [6]. The traditional efficacy of *T. sinensis* is closely associated with a variety of phytochemical constituents. Previous phytochemical investigations on this plant have revealed that the secondary metabolites include triterpenoids, sesquiterpenoids, diterpenoids, sterols, phenols, flavonoids and phenylpropanoids [7,8]. Among the phytochemical constituents, triterpenoids are known to be the main constituents, such as limonoid, *apo*-tirucallane and tirucallane [9]. It is important to quickly characterize the natural products in these complex plant extracts. Mass spectrometry is a powerful tool for analyzing chemical compositions. Understanding the cracking laws of compounds from *T. sinensis* is important for the discovery of active substances with novel structures [10]. Modern studies have also reported that *T. sinensis* possesses various pharmacological activities, including antidiabetic, antidiabetic nephropathy, antioxidant, anti-inflammatory, antitumor, hepatoprotective, antiviral, antibacterial and other biological activities [11,12,13] (Figure 1). Together, these findings have provided many new insights and a strong scientific basis for supporting its practical use in medical situations.

In this review, we compile the progress on phytochemical studies over the past few decades, with all the elucidated compounds listed. The biological characterizations of the extracts and compounds isolated from *T. sinensis* plant are also discussed. Therefore, this review will provide a guide for the full utilization of these plants for new drug development and pharmaceutical applications through a comprehensive understanding of the development status of *T. sinensis*.

## 2. Traditional Uses

*T. sinensis*, a deciduous woody plant native to Eastern and Southeastern Asia, is used as a vegetable source in China and Malaysia and as animal fodder in India [14,15]. The *T. sinensis* plant, a unique tree species in China, is popularly known as “Xiang Chun”, “Chinese *toon*” or “Chinese mahogany” and has a long history in medicine, with wide uses and rich sources [16,17].

*T. sinensis* has been widely used in traditional Chinese medicine (TCM), the effects of which, including heat-clearing, diuresis and detoxification, were known in ancient times. All parts of this plant, including the leaves, seeds, barks, buds and pericarps, have been traditionally used in folk medicine to treat various diseases. *T. sinensis* leaves taste bitter and flat. Their summer dampness-dispelling, detoxification and insecticidal effects are used to treat the spleen and stomach channel for summer-dampness injury, nausea, vomiting, loss of appetite and other symptoms [18]. The barks of *T. sinensis* are bitter, astringent and slightly cold and are used to clear heat and dispel dampness to treat an astringent intestine by stopping bleeding, treating band disease and killing insects. The barks are used for diarrhea, dysentery, intestinal wind, blood stools, disc leakage and other symptoms [19]. *T. sinensis* seeds taste bitter and warm. Their wind-dispelling, cold and analgesic effects are used to treat the lung, liver and large intestine meridian for external wind cold, rheumatism, stomach pain and other diseases [20,21].

*T. sinensis* is used as a medicinal plant in traditional medicine. Appropriate amounts of *T. sinensis* buds and vinegar are weighed. *T. sinensis* buds are soaked in vinegar and then brewed in boiling water into *T. sinensis* soups to treat colds, which are administered as one dose daily divided into three. *T. sinensis* leaves (50 g) are weighed, washed and mashed and then mixed with rice vinegar or yellow rice wine to treat oral and tongue sores, which are administered as one dose daily divided into two. *T. sinensis* leaves, garlic and a small amount of salt are weighed and mashed together to form a mud, which is applied to an affected area to treat sores and swelling twice a day for 1–2 h each time. *T. sinensis* leaves (20 g), “Jiao Sanxian” (20 g), *Agastache rugosa* (10 g) and *Nelumbo nucifera* seeds (15 g) are weighed, boiled in water to remove the residue and extract the juice and used to treat spleen and stomach weaknesses and abdominal distention. This is administered as one dose daily divided into two. *T. sinensis* root barks (60 g) are weighed, boiled in water to remove the residue and extract the juice, with an appropriate amount of brown sugar added to the juice. This is administered as one dose daily divided into three for the treatment of white band disease. *T. sinensis* seeds (30 g) are cooked with pork or mutton and used once a week to treat rheumatic joint pain [22].

## 3. Phytochemical Constituents

A phytochemical investigation is a critical step in understanding the therapeutic potential of medicinal plants. As a botanical source of bioactive compounds, *T. sinensis* has been the subject of extensive research. To date, 206 compounds have been isolated and characterized from *T. sinensis*, including triterpenoids (**1**–**133**), sesquiterpenoids (**134**–**135**), diterpenoids (**136**–**142**), sterols (**143**–**147**), phenols (**148**–**167**), flavonoids (**168**–**186**), phenylpropanoids (**187**–**192**) and others (**193**–**206**). The chemical structures of these compounds are illustrated in Table 1.

### 3.1. Triterpenoids

**Figure 2:**
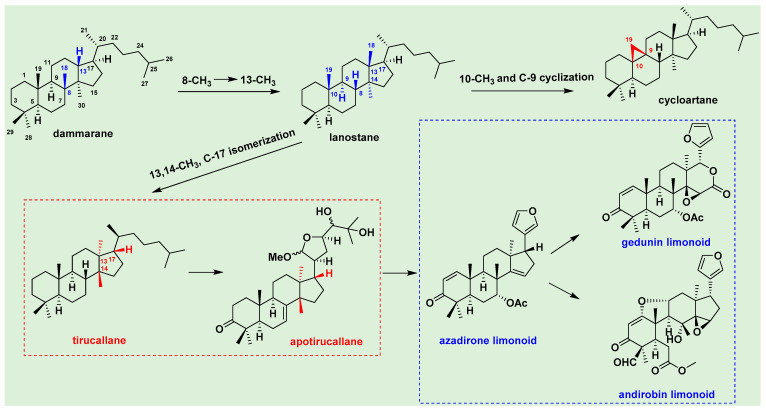
Main skeleton structures of tetracyclic triterpenoids from *T. sinensis*.

Triterpenoids are a class of terpenoids. The basic nucleus of a terpenoid is composed of 30 carbon atoms. Triterpenoids exist in plants in free form or as glycosides or esters combined with sugars. Triterpenoids are the main components of *T. sinensis*. A total of 133 triterpenoids have been isolated from various parts of *T. sinensis*, including dammarane, tirucallane, *apo*-tirucallane, limonoids, cycloartane and other triterpenoids. The most abundant tetracyclic triterpenoids in *T. sinensis* include dammarane, tirucallane, *apo*-tirucallane and limonoid triterpenoids [47]. Their structural correlations are shown in Figure 2.

### 3.2. Dammarane Triterpenoids

**Figure 3:**
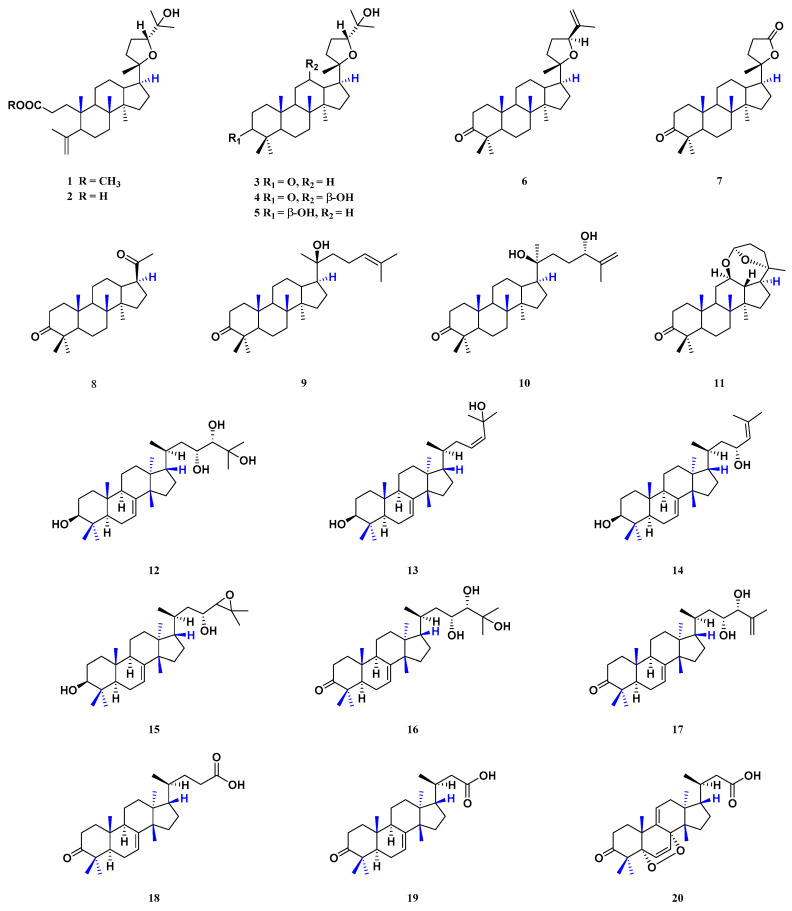
Structure of dammarane and tirucallane triterpenoids from *T. sinensis*.

Dammarane triterpenoids derived from the “full chair” conformation of epoxy-squalene are characterized by a C-8 angular methyl group with a *β*-configuration. In addition, the C-13 position has a *β*-H configuration. The C-10 position has a *β*-CH_3_ configuration. The C-17 position has *β*-side chains. C-20 has an *R* or *S* configuration. At present, eleven dammarane triterpenoids (**1**–**11**) have been isolated from the stem barks of *T. sinensis* [23], including methyl shoreate (**1**), shoreic acid (**2**), ocotillone (**3**), (20*S*, 24*R*)-epoxydammarane-12, 25-diol-3-one (**4**), (20*S*, 24*R*)-epoxydammarane-3*β*, 25-diol-marane-3*β*, 25-diol (**5**), richenone (**6**), cabralealactone (**7**), hollongdione (**8**), 20-hydroxy-24-dammaren-3-one (**9**), (20*S*, 24*S*)-dihydroxydammar-25-en-3-one (**10**) and cylindrictone D (**11**) (Figure 3).

### 3.3. Tirucallane Triterpenoids

Tirucallane triterpenoids have a basic parent nucleus of cyclopentane, the A/B, B/C and C/D rings of which have a trans configuration. Tirucallane triterpenoids, in general, have five methyl groups and a side chain composed of eight carbon atoms on the C-17 position of the parent nucleus. That is, the C-4 position has two methyl groups. The C-10 and C-14 positions have one methyl group each (10*β* and 14*β*, respectively), and another methyl group is connected to the C-13 position (13*α*). The C-17 side chain is an *α* configuration. At present, nine tirucallane triterpenoids (**12**–**20**) have been isolated from the barks, stem barks and seeds of *T. sinensis*. Three tirucallane triterpenoids (**12**, **16** and **17**) have been isolated from the barks of *T. sinensis* [24,26]. Three tirucallane triterpenoids (**13**–**15**) have been isolated from the seeds of *T. sinensis* [25]. Three tirucallane triterpenoids (**18**–**20**) have been isolated from the stem barks of *T. sinensis* [23] (Figure 3).

### 3.4. Apo-Tirucallane Triterpenoids

**Figure 4:**
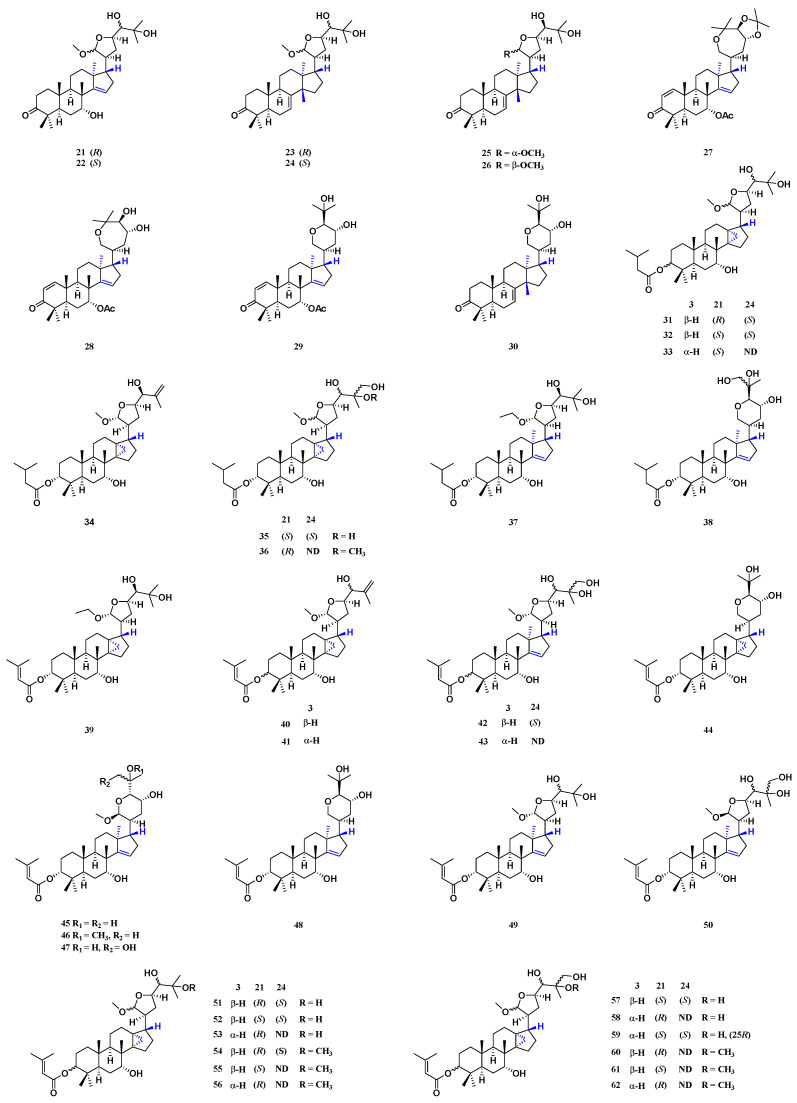
Structure of *apo*-tirucallane triterpenoids from *T. sinensis*.

Tirucallane triterpenoids are thought to be the precursors of *apo*-tirucallane triterpenoids. *Apo*-tirucallane triterpenoids are parent-nucleus D rings with the Wagen–Meerwein rearrangement. This rearrangement results in the formation of double bonds at positions C-14 and C-15. The α side chains connected at positions C-17 may exhibit structural changes (such as the branched chain, ring formation, hydroxylation, epoxidation and other structural changes), which are, in general, tetrahydrofuran rings, tetrahydropyran rings and hepta-membered oxygen-containing rings. Forty-two *apo*-tirucallane triterpenoids (**21**–**62**) have been isolated from the barks, seeds, leaves, stems and pericarps of *T. sinensis*. Thirteen *apo*-tirucallane triterpenoids (**21**–**24**, **27**–**30**, **38**, **43** and **48**–**50**) have been isolated from the barks of *T. sinensis* [24]. Twenty-six *apo*-tirucallane compounds (**31**–**36**, **40**–**47** and **51**–**62**) have been isolated from the seeds, leaves and stems of *T. sinensis* [28]. Seven *apo*-tirucallane triterpenoids (**25**–**26**, **34**, **37**, **39**, **41** and **43**) have been isolated from the pericarps of *T. sinensis* [1,15,27] (Figure 4).

### 3.5. Limonoid Triterpenoids

**Figure 5:**
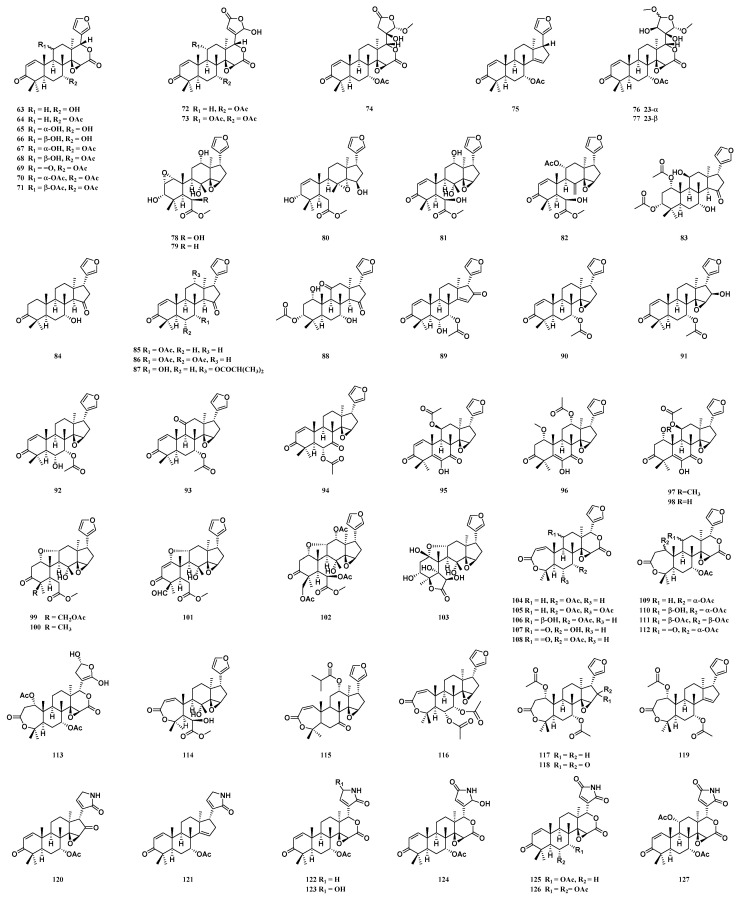
Structure of limonoid triterpenoids from *T. sinensis*.

Limonoid triterpenoids are a class of highly oxidized compounds with a skeleton of 4,4,8-trimethyl-17-furanosteroid or one of its derivatives. Biogenetically, limonoids are derived from the degradation of Δ^7^-tinucallol or Δ^7^-euphol by the loss of four carbon atoms at the end of the C-17 side chain. Hence, this class of compounds is also called “tetranormo-triterpenes”. At present, sixty-five limonoid triterpenoids (**63**–**127**) have been isolated from the barks, root barks, leaves and buds of *T. sinensis*. Fourteen limonoid triterpenoids (**63**–**72**, **75**, **123**, **125** and **126**) have been isolated from the barks of *T. sinensis* [24,26,29]. Sixteen limonoid triterpenoids (**73**–**74**, **76**–**78**, **104**, **111**, **116**–**122**, **124** and **127**) have been isolated from the root barks of *T. sinensis* [30,33]. Nineteen limonoid triterpenoids (**79**–**82**, **99**–**103**, **105**–**110** and **112**–**115**) have been isolated from the leaves of *T. sinensis* [31,34,35]. Sixteen limonoid triterpenoids (**83**–**98**) have been isolated from the leaves and buds of *T. sinensis* [32] (Figure 5).

### 3.6. Cycloartane and Other Triterpenoids

**Figure 6:**
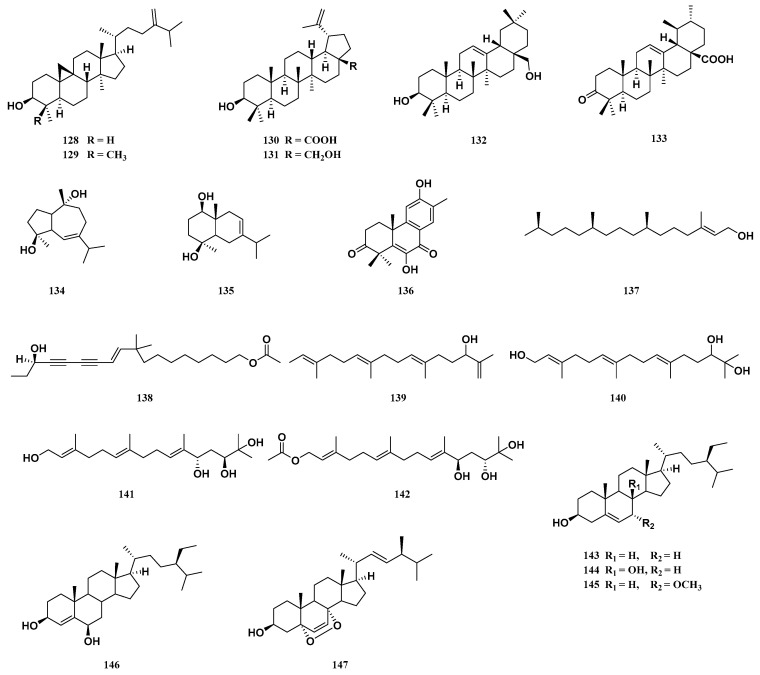
Structure of cycloartane triterpenoids, other triterpenoids, sesquiterpenoids, diterpenoids and sterols from *T. sinensis*.

Two cycloartane triterpenoids (**128** and **129**) have been isolated from the pericarps of *T. sinensis* [1,15,27]. Two lupinane triterpenoids (**130** and **131**) and one oleanane triterpenoid (**132**) have been isolated from the barks of *T. sinensis* [26]. One ursane triterpenoid (**133**) has been isolated from the roots of *T. sinensis* [36] (Figure 6).

### 3.7. Sesquiterpenoids and Diterpenoids

Sesquiterpenoids and diterpenoids are typically synthesized by polymerizing three to four molecules of isoprene. Sesquiterpenoids are natural terpenoids containing 15 carbon atoms. At present, two sesquiterpenoids (**134** and **135**) have been isolated from the pericarps of *T. sinensis* [1,27]. Diterpenoids are terpenoids containing four isoprene units. They are natural products with complex and diverse structures and important biological activities. Seven diterpenoids (**136**–**142**) have been isolated from *T. sinensis*. Two diterpenoids (**136** and **138**) have been isolated from the barks of *T. sinensis* [26,37]. Three diterpenoids (**137**,**139** and **140**) have been isolated from the leaves of *T. sinensis* [34]. Two diterpenoids (**141** and **142**) have been isolated from the seeds of *T. sinensis* [25] (Figure 6).

### 3.8. Sterols

Sterols are derivatives of a hydrogenated benzene ring system. They are important active substances that are widely present in organisms. Five sterols (**143**–**147**) have been isolated from the pericarps of *T. sinensis* [1,27]. Compound **143** has also been isolated from the barks and roots of *T. sinensis* [33,38] (Figure 6).

### 3.9. Phenols

**Figure 7:**
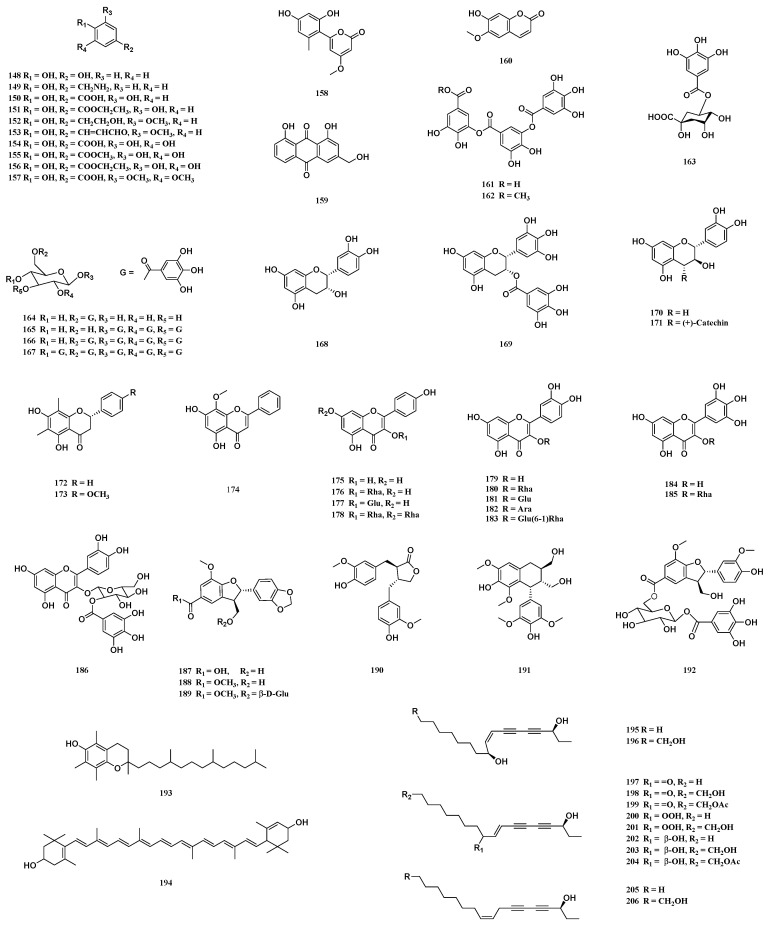
Structure of phenols, flavonoids, phenylpropanoids and other compounds from *T. sinensis*.

Phenols are naturally occurring metabolites found widely in plants. They have diverse pharmacological activities. Various phenolic compounds are distributed in different parts of *T. sinensis*. The contents of phenolic acid and its derivatives are relatively high. Twenty phenols (**148**–**167**) have been isolated from various parts of *T. sinensis*. Twelve compounds (**148**–**156**, **161**–**162** and **167**) have been isolated from the pericarps of *T. sinensis* [1,27]. Compounds **155** and **167** have also been isolated from the young leaves of *T. sinensis* [39]. Four phenols (**157**–**160**) have been isolated from the roots of *T. sinensis* [33]. Compound **163** has been isolated from the leaves of *T. sinensis* [12]. Three compounds (**164**–**166**) have been isolated from leaves and shoots of *T. sinensis* [39] (Figure 7).

### 3.10. Flavonoids

In general, a “flavonoid” refers to a compound formed by connecting two phenyl rings and a heterocyclic ring. That is, the general structure of flavonoids is a 15-carbon skeleton of C_6_-C_3_-C_6_. Flavonoids exist in almost all green plants (mainly in higher plants) and have a wide range of biological activities. The subclassification of flavonoids with different aglycones can be divided into flavan-3-ols, flavanones, flavones and flavonols, all of which have been isolated from *T. sinensis*. Nineteen flavonoids (**168**–**186**) have been isolated from various parts of *T. sinensis*. One flavan-3-ols (**168**) and two flavanones (**172** and **173**) have been isolated from the stems of *T. sinensis* [40]. One flavan-3-ols (**169**) and one flavonol (**186**) have been isolated from the leaves of *T. sinensis* [12,40]. Two flavan-3-ols (**170** and **171**) have been isolated from the leaves and wood of *T. sinensis* [42]. One flavone (**174**) and two flavonols (**184** and **185**) have been isolated from the barks of *T. sinensis* [38]. Five flavonols (**175**–**177**, **179** and **180**) have been isolated from the young leaves of *T. sinensis* [39,41]. Three flavonols (**180**–**182**) have been isolated from the pericarps of *T. sinensis* [1,25,39]. One flavonol (**183**) has been isolated from the leaves and shoots of *T. sinensis* [39] (Figure 7).

### 3.11. Phenylpropanoids and Other Compounds

Phenylpropanoids are a group of naturally occurring organic compounds with one or several C_6_-C_3_ units in the basic parent nucleus. They are present widely in higher plants. Six phenylpropanoid compounds (**187**–**192**) have been isolated from the leaves, root barks and pericarps of *T. sinensis*. Two phenylpropanoid compounds (**187** and **189**) have been isolated from the leaves of *T. sinensis* [44]. Three phenylpropanoid compounds (**188**, **190** and **191**) have been isolated from the root barks of *T. sinensis* [33]. Two phenylpropanoid compounds (**188** and **192**) have been isolated from the pericarps of *T. sinensis* [1,27] (Figure 7). Two compounds (**193** and **194**) have been isolated from the leaves of *T. sinensis* [45]. Twelve compounds (**195**–**206**) have been isolated from the root barks of *T. sinensis* [46] (Figure 7).

Based on literature findings, we summarized the chemical constituents isolated and purified from different parts of *T. sinensis*, which are helpful in identifying the active constituents of different medicinal parts and provide a reference for subsequent pharmacodynamics research.

## 4. Compound Cracking Laws

Molecules can undergo a variety of ionizations in ion sources, and the same molecule can produce a variety of ions. Many ion peaks can also be seen from the mass spectrum, and most of the ion peaks formed according to the self-cracking laws of the compounds.

**Figure 8:**
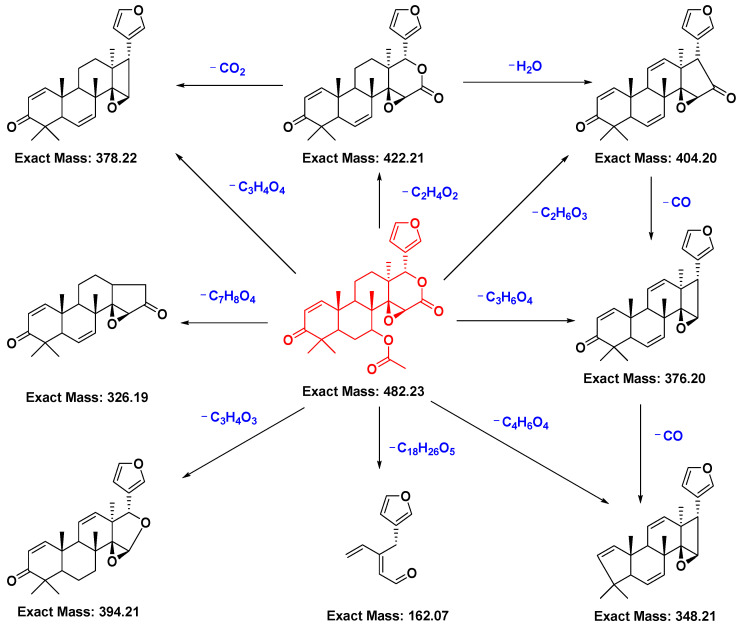
The proposed fragmentation pathway of gedunin (**64**) [48].

Limonoid-type triterpenoids are the main chemical constituents of *T. sinensis*. Summarizing the cracking laws of these compounds by mass spectrometry (MS) for analyses of the chemical constituents of *T. sinensis* is important. The dissociation behaviors of limonoids upon high-resolution electrospray ionization–tandem mass spectrometry (HR-ESI-MS/MS) have been proposed [48]. In this review article, the possible fragmentation pathways of gedunin (**64**) (typical limonoid-type triterpenoid) were deduced. In positive-ion mode, gedunin was detected as the [M + H]^+^ ion at *m*/*z* 483.2369 (C_28_H_35_O_7_, Cal. 483.2377). In its MS/MS spectrum, common ions at *m*/*z* 423.2162 (C_26_H_31_O_5_), 405.2055 (C_26_H_29_O_4_), 395.2211 (C_25_H_31_O_4_), 379.2264 (C_25_H_31_O_3_), 377.2099 (C_25_H_29_O_3_), 327.1951 (C_21_H_27_O_3_) and 161.0594 (C_10_H_9_O_2_), assigned as [M + H − C_2_H_4_O_2_]^+^, [M + H − C_2_H_6_O_3_]^+^, [M + H − C_3_H_4_O_3_]^+^, [M + H − C_3_H_4_O_4_]^+^, [M + H − C_3_H_6_O_4_]^+^, [M + H − C_7_H_8_O_4_]^+^ and [M + H − C_18_H_26_O_5_]^+^, respectively, were observed (Figure 8). Neutral losses of C_2_H_4_O_2_, H_2_O, CO and CO_2_ were the main fragmentation patterns for limonoids in positive-ion mode. In addition, an identical characteristic ion at *m*/*z* 161.0594 (C_10_H_9_O_2_) was found in the MS/MS spectra of the four limonoids, which played an important part in metabolite identification.

Cycloartane-type triterpenoids are another type of triterpenoid from *T. sinensis*. The molecular weight of cycloeucalenol (**128**) and 24-methylenecycloartanol (**129**) was confirmed by their pseudo-molecular ions: the [M − H_2_O + H]^+^ of cycloeucalenol (*m*/*z* 409) and 24-methylenecycloartanol (*m*/*z* 423), respectively. This confirmation was made using normal-phase liquid chromatography–mass spectrometry operating in atmospheric pressure chemical ionization mode. The protonated molecular ions [M + H]^+^ of cycloeucalenol (*m*/*z* 427) and 24-methylenecycloartanol (*m*/*z* 441) were very abundant. The spectrum of cycloeucalenol (**128**) showed fragment ions at *m*/*z* 426 [M]^+^, 411 [M − CH_3_]^+^, 408 [M − H_2_O]^+^, 393 [M − CH_3_ − H_2_O]^+^, 353 [M − C_3_H_7_ − 2CH_3_ − H_2_O]^+^ and 300 [M − C_7_H_13_ − CH_2_ − CH_3_]^+^, which were tentatively identified using gas chromatography–mass spectrometry. The fragment ions of 24-methylenecycloartanol (**129**) were at 440 [M]^+^, 425 [M − CH_3_]^+^, 422 [M − H_2_O]^+^, 407 [M − CH_3_ − H_2_O]^+^, 397 [M − C_3_H_7_]^+^, 379 [M − C_3_H_7_ − H_2_O]^+^, 315 [M − C_9_H_17_]^+^, 300 [M − C_9_H_17_ − CH_3_]^+^, 285 [M − C_9_H_17_ − 2CH_3_]^+^ and 203 [M − C_9_H_17_ − 5CH_3_]^+^ [49].

Phenols and flavonoids are the main secondary metabolites of *T. sinensis*. In negative-ion mode, gallic acid (**154**) was detected as the [M − H]^−^ ion at *m*/*z* 169.0137 (C_7_H_6_O_5_). In its MS/MS spectrum, common ions were at *m*/*z* 107.0115, 125.0238 and 140.0717. Methyl gallate (**155**) was detected as the [M − H]^−^ ion at *m*/*z* 183.0302 (C_8_H_8_O_5_). In its MS/MS spectrum, common ions were at *m*/*z* 106.0090, 124.016, 125.0211, 151.0040 and 168.0074. Ethyl gallate (**156**) was detected as the [M − H]^−^ ion at *m*/*z* 197.0457 (C_9_H_10_O_5_). In its MS/MS spectrum, common ions were at *m*/*z* 78.01, 124.0162, 125.0237, 151.0038 and 169.014 [50]. In brief, an identical characteristic ion at *m*/*z* 125.02 was found in the MS/MS spectrum, which played an important part in the metabolite identification of gallic acid and its derivatives.

In negative-ion mode, kaempferol (**175**, KAE) was detected as the [M − H]^−^ ion at *m*/*z* 285.0405 (C_15_H_10_O_6_). In its MS/MS spectrum, common ions were at *m*/*z* 107.0136, 159.0488, 163.0048, 255.0297 and 285.0388 [51]. In negative-ion mode, astragalin (**177**, kaempferol 3-*O*-*β*-D-glucoside) was detected as the [M − H]^−^ ion at *m*/*z* 447.0933 (C_21_H_20_O_11_). In its MS/MS spectrum, common ions were at *m*/*z* 125.0271, 163.0381, 283.1305, 285.0368, 295.0438, 357.0590 and 447.0926 [51]. In negative-ion mode, quercetin (**179**) was detected as the [M − H]^−^ ion at *m*/*z* 301.0354 (C_15_H_10_O_7_). In its MS/MS spectrum, common ions were at *m*/*z* 83.0210, 93.0366, 107.0121, 109.0269, 121.0300, 149.0260, 151.0027, 163.0004, 178.9969, 193.0103, 273.0378 and 301.0334 [51]. In negative-ion mode, rutin (**183**) was detected as the [M − H]^−^ ion at *m*/*z* 609.1444 (C_27_H_30_O_16_). In its MS/MS spectrum, common ions were at *m*/*z* 61.9878, 151.0029, 301.0344, 343.0444, 389.1586, 463.0868 and 571.1987 [50]. In summary, an identical characteristic ion at *m*/*z* 163 was found in the MS/MS spectrum, which might have an important role in the metabolite identification of flavonoids and their derivatives.

## 5. Pharmacological Activities

*T. sinensis*, a well-known medicinal herb, has been traditionally used for treating various diseases. Our review of the pharmacological activities of *T. sinensis* showed that the bioactive properties included antidiabetic, antidiabetic nephropathy, antioxidant, anti-inflammatory, antitumor, hepatoprotective, antiviral and antibacterial, and immunopotentiation effects on the male reproductive system and other activities. Detailed information of *T. sinensis* is shown in Table 2. These properties of *T. sinensis* could help us to understand its pharmacological activities. They also encourage us to use it without hesitation as a treatment for related diseases.

### 5.1. Antidiabetic Activity

Diabetes mellitus (DM) is a chronic metabolic disease characterized by hyperglycemia. In recent decades, the hypoglycemic effects of *T. sinensis* have attracted increasing attention. All parts of *T. sinensis* have different degrees of inhibition of DM. The non-polar extracts of *T. sinensis* leaves (TSLs) prepared using supercritical-CO_2_ fluid have been shown to prevent the progression of DM and liver fibrosis, increase triglyceride levels and decrease adiponectin levels in low-dose streptozotocin (STZ)-induced mice with type-2 diabetes mellitus (T2DM) [52]. Hence, TSL non-polar extracts might contain active ingredients to prevent T2DM [52]. The effects of TSL water extracts on alloxan-induced diabetic Long-Evans rats have been studied. After administration of TSL extract or gallic acid (**154**), the mRNA and protein expression of glucose transporter 4 (GLUT4) increased significantly in rats suffering from DM. Therefore, TSLs have hypoglycemic effects, and the mechanism of action involves an increase in the insulin level mediating the action of GLUT4 in fat [53]. Cellular glucose uptake with a combination of TSL water extracts and insulin has been found to be inhibited significantly by treatment of 3T3-L1 adipocytes with cycloheximide (inhibitor of protein synthesis) and calphostin C (inhibitor of protein kinase C) in normal-, medium- and high-glucose media [54]. The anti-DM effects and mechanism of action of 95% ethanol (EtOH) extracts from TSLs have also been studied in vitro and in vivo. TSL EtOH extracts have been shown to stimulate glucose uptake via adenosine monophosphate-activated protein kinase (AMPK) activation in skeletal muscles, promote the expression of peroxisome proliferator-activated receptor-gamma and normalize adiponectin expression in adipose tissues, thereby ameliorating insulin resistance [55].

Rutin (**183**) from TSL water extracts can improve glucose uptake in C57BL/6 mice with insulin-resistant T2DM by increasing insulin-dependent receptor kinase (IRK) activity [56]. Quercetin (**179**), a flavonoid isolated from TSL ethyl acetate (EtOAc) extracts, can reduce hyperglycemia induced by the consumption of a high-carbohydrate/high-fat diet (HFD) and alloxan in mice suffering from DM. Quercetin (**179**) significantly inhibits the activation of p65/nuclear factor-kappa B (NF-κB) and the extracellular signal-regulated kinase 1/2/mitogen-activated protein kinase (ERK1/2/MAPK) pathways, as well as the levels of caspase-9 and caspase-3 in the liver tissue of mice with DM [57]. These actions can reduce the risk of DM and its secondary complications by lessening oxidative stress in the liver [57].

### 5.2. Antidiabetic Nephropathy Activity

**Figure 9:**
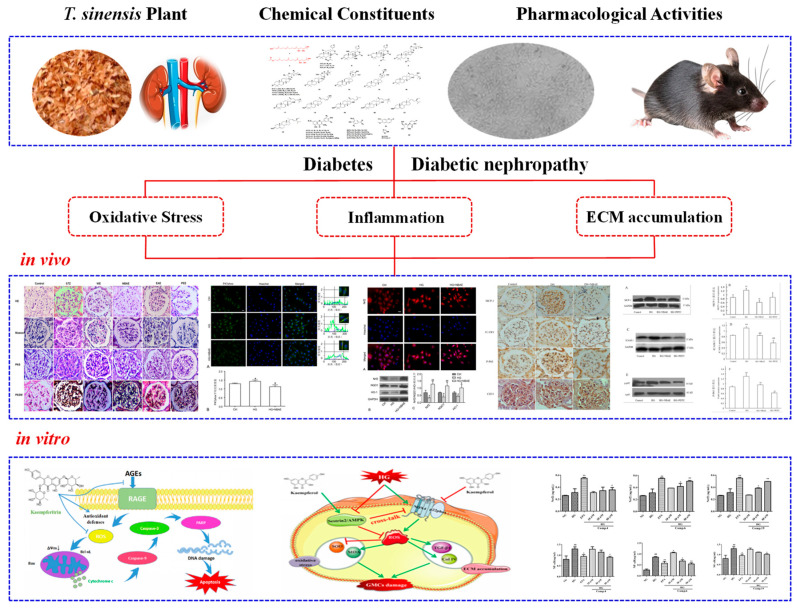
Antidiabetic and antidiabetic nephropathy activities of extracts or compounds from *T. sinensis*.

The petroleum-ether extracts of *T. sinensis* seeds could reduce the blood glucose level, urinary albumin level, serum creatinine level and urea nitrogen level, as well as indices of renal function and oxidative stress. Renal abnormalities could be improved in rats suffering from diabetic nephropathy (DN). Protein expression of transforming growth factor-*β*1 (TGF-*β*1), collagen IV (Col IV) and connective tissue growth factor (CTGF) could be reduced using the petroleum-ether extracts of *T. sinensis* seeds, and the petroleum-ether extracts of *T. sinensis* seeds have been shown to have protective effects on rats DN by inhibiting oxidative stress and protein expression of TGF-*β*1, Col IV and CTGF [58]. The *n*-butanol extracts of *T. sinensis* seeds (NBAE) could significantly reduce the blood glucose level, urinary albumin level, serum creatinine level and urea nitrogen level, as well as the indices of kidney function and oxidative stress. NBAE could increase the activities of total antioxidant capacity (T-AOC), superoxide dismutase (SOD), glutathione peroxidase (GSH-Px) and catalase (CAT), and reduce the level of malondialdehyde (MDA) in the serum of rats with STZ-induced DN, showing significant antioxidant activity in vivo. NBAE have been found to inhibit the expression of TGF-*β*1, Col IV and CTGF protein in rats with STZ-induced DN, showing protective effects on the kidney in these animals [59]. High glucose (HG) induces oxidative stress injury after stimulating glomerular mesangial cells (GMCs). This action leads to an increased reactive oxygen species (ROS) level, decreased nitric oxide (NO) level, increased expression of p47phox and decreased expression of nuclear factor erythroid 2-related factor 2 (Nrf2) and its downstream proteins NAD(P)H quinone oxidoreductase 1 (NQO1) and heme oxygenase-1 (HO-1). NBAE can significantly increase the expression of Nrf2, NQO1 and HO-1, thereby inhibiting HG-induced ROS elevation, inhibiting p47phox expression and stabilizing NO content [60]. Compared with the DN group, in the DN+NBAE group, the blood glucose level was reduced significantly and injury was alleviated. Otherwise, levels of monocyte chemoattractant protein-1 (MCP-1), intercellular adhesion molecule 1 (ICAM-1) and phosphorylated-p65 were reduced. In vitro, NBAE decreased the expression of MCP-1 and ICAM-1 significantly, which was similar to the effect elicited by treatment with a blocker of NF-κB p65 [61] (Figure 9).

GMCs were cultured and induced by advanced glycosylation end products (AGEs) to simulate DN in vitro. The mechanism of action of kaempferitrin (**178**, KM) from *T. sinensis* seeds to protect GMCs from AGE-induced damage was investigated. KM could increase SOD activity, reduce the level of MDA, inhibit ROS production and protect against oxidative stress in AGE-induced GMCs. These findings suggest that KM might be a drug for treating DN in the future [43]. KAE (**175**) significantly reduced levels of ROS, NADPH oxidase (NOX) and MDA and enhanced SOD activity in HG-induced GMCs. The production of TGF-*β*1, Col IV, NOX4 and p22phox was also inhibited by KAE treatment. In addition, KAE increased the expression of sestrin2 and AMPK in HG-induced GMCs [62]. Three apotirucallane-type triterpenoids, toonasinensin B (**39**), 21*β*-*O*-methylmelianodiol (**26**) and 21*α*-*O*-methylmelianodiol (**25**), from the pericarps of *T. sinensis*, could increase SOD activity significantly and reduce the levels of MDA and ROS, thereby preventing DN by reducing oxidative stress in GMCs cultured under HG conditions [15]. Additionally, in this model, the cytotoxicity and polyol pathway inhibitory activities of active constituents from the pericarps of *T. sinensis* were evaluated. Toonasinensin B (**39**), toonasinensin D (**41**), 21*β*-*O*-methylmelianodiol (**26**) and 21*α*-*O*-methylmelianodiol (**25**) had good inhibitory effects on GMCs. Moreover, it was shown that toonasinensin B (**39**), 21*β*-*O*-methylmelianodiol (**26**) and 21α-*O*-methylmelianodiol (**25**) inhibited the production of NADPH and sorbitol in HG-induced GMCs for the first time. These compounds could be developed for the treatment of DN [27]. Two acyclic diterpenoids (**141** and **142**) were isolated from the seeds of *T. sinensis*. They could significantly upregulate Nrf2/HO-1 expression and reduce the expression of NF-κB, tumor necrosis factor-alpha (TNF-α) and interleukin-6 (IL-6), thereby improving oxidative stress in HG-induced GMCs [25] (Figure 9).

### 5.3. Antioxidant Activity

The *α*,*α*-diphenyl-*β*-pricryl-hydrazyl (DPPH) radical scavenging test showed that the DPPH free-radical scavenging activities of extracts of the leaves, roots and barks of *T. sinensis* were concentration-dependent, and the half-maximal inhibitory concentrations (IC_50_) were 2.09 × 10^−1^, 2.85 × 10^−1^ and 2.77 × 10^−1^ mg/mL, respectively. These extracts could also reduce the accumulation of amyloid *β*-protein, thiobarbituric acid-reactive substances (TBARS) and cognitive deterioration in mice and increase the activities of SOD, CAT and GSH-Px to promote the antioxidant defense system. The compounds of *T. sinensis* extracts could delay the aging process in mice, which merits further study [63]. The antioxidant activities of TSL acetone extracts, including oxygen radical absorption capacity (ORAC), peroxyl radical scavenging capacity (PSC) and cellular antioxidant activity (CAA), were evaluated. Anti-proliferative activities against human liver cancer (HepG2) cells were assessed using the methylene-blue assay. TSL acetone extracts possessed significant antioxidant properties and anti-proliferative effects against HepG2 cells in vitro [64]. TSL aqueous extracts and gallic acid (**154**) treatment significantly inhibited ROS generation and MDA formation in 2,2′-azo-bis (2-amidinopropane) hydrochloride (AAPH)-stimulated human umbilical vein endothelial cells. Furthermore, pretreatment with TSL aqueous extracts/gallic acid significantly augmented AAPH-depleted SOD/CAT activity in endothelial cells. However, AAPH-induced Bax/B-cell lymphoma-2 (Bcl-2) dysregulation was reversed significantly by pretreatment with TSL aqueous extracts/gallic acid. Therefore, *T. sinensis* might have antioxidant effects to protect endothelial cells from oxidative stress [65]. TSL also showed that the aqueous extracts and gallic acid (**154**) had effective antioxidant activity against various oxidative systems in vitro, including the scavenging of free-radicals and superoxide anion radicals, total reducing power and metal chelation. Furthermore, AAPH-induced oxidative hemolysis, lipid peroxidation and a decline in SOD activity in human erythrocytes were prevented by TSL extracts and gallic acid (**154**). In conclusion, TSL aqueous extracts and gallic acid (**154**) have antioxidant properties [66].

The IC_50_ values of 2,2′-azinobis (3-ethylbenzothiazoline-6-sulphonic acid ammonium salt) (ABTS) and DPPH free-radical scavenging activities of seven flavonoids and methyl gallate (**155**) extracted from the 70% methanol extracts of *T. sinensis* buds were 1.4–3.6 and 2.6–671.0 μg/mL, respectively, indicating that these compounds showed significant antioxidant activity [67]. 1,2,3,4,6-Penta-*O*-galloyl-*β*-D-glucose (**167**) and ethyl gallate (**156**) were obtained from the young leaves of *T. sinensis* by establishing a liquid–liquid refined extraction-guided bioassay. The EC_50_ (the concentration for 50% of maximal effect) values of ethyl gallate (**156**) scavenging ABTS and DPPH were 4.46 ± 0.05 and 7.61 ± 0.13 μg/mL, respectively, and those of 1,2,3,4,6-penta-*O*-galloyl-*β*-D-glucose (**167**) scavenging ABTS and DPPH were 12.90 ± 0.16 and 16.29 ± 0.20 μg/mL, respectively, indicating that they were good antioxidants [68]. Four chemical-induced oxidative models were applied in the previous study, including DPPH free-radical scavenging assay, phenazine methosulphate (PMS) nicotinamide adenine dinucleotide (NADH) PMS-NADH-NBT superoxide anion scavenging assay, FeCl_3_-K_3_Fe (CN)_6_ reducing power assay and FeCl_2_-FerroZine metal chelation assay. Quercetin (**179**), kaempferol-3-*O*-α-L-rhamopyranoside (**176**), astragalin (**177**), KAE (**175**), methyl gallate (**155**), ethyl gallate (**156**) and 1,2,3,4,6-penta-*O*-galloyl-*β*-D-glucopyranose (**167**) isolated from the young leaves of *T. sinensis* had several significant antioxidant properties [41]. Twelve limonoids were isolated from TSLs. Their antioxidant evaluation showed that toonasinenine D (**103**), E (**82**), G (**81**), H (**80**), I (**114**) and J (**115**) had significant anti-radical activities compared with the radicals tested using DPPH and ABTS. Toonasinenine D (**103**) seemed to possess higher anti-radical activities on ABTS but lower scavenging activity on DPPH than other compounds [31].

### 5.4. Anti-Inflammatory Activity

TSL water extracts could upregulate the expression of HO-1 and downregulate the expression of TNF-*α* to inhibit the lipopolysaccharide (LPS)-induced inflammatory response from macrophages [69]. TSL aqueous extracts increased the level of total GSH and the ratio of GSH/glutathione oxide (GSSG) in RAW264.7 cells treated with LPS but decreased the levels of GSSG, total NO, nitrate, nitrite, MDA and superoxide anion. TSL water extracts reversed the effects of LPS-induced cytokines, including IL-6 and IL-10, to modulate autophagy during inflammation [70]. The adventitious shoot extracts of *T. sinensis* showed good anti-inflammatory activity on LPS-treated RAW 264.7 cells and *Propionibacterium acnes*-treated HaCaT cells. Hence, the adventitious shoot extracts of *T. sinensis* could be used as a drug for the treatment of inflammatory skin diseases. The effects were regulated by suppression of the MAPK pathway [71]. TSL aqueous extracts possessed effective anti-inflammatory features, including the suppression of LPS-induced NO production, as well as TNF-α secretion and protein expression of inducible nitric oxide synthase (iNOS) in BV-2 microglial cells without cytotoxicity. The results indicated that TSL aqueous extracts could inhibit the inflammatory response of microglia in neurodegenerative diseases [72]. Polyphenols extracted from *T. sinensis* seeds alleviated 6-hydroxydopamine-induced neuroinflammation by inhibiting the p38 MAPK signaling pathway in a rat model of Parkinson′s disease [73].

**Figure 10:**
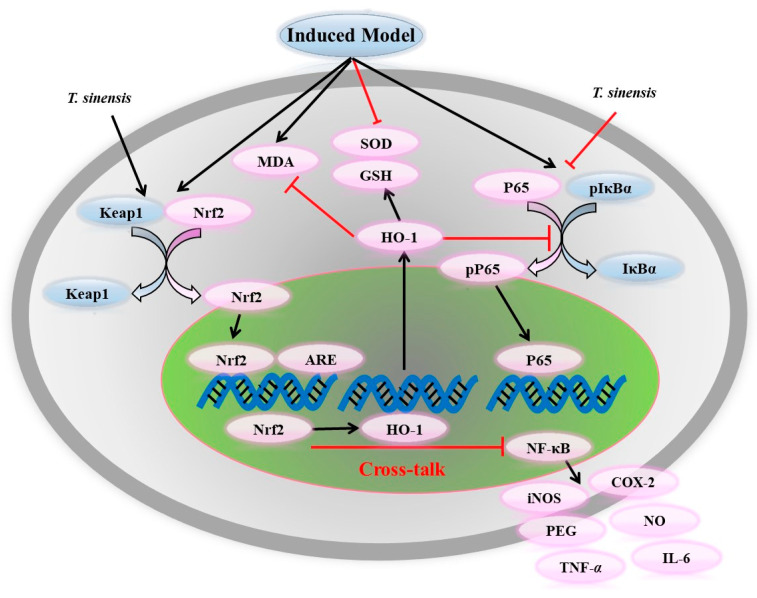
Antioxidant and anti-inflammatory activities of extracts or compounds from *T. sinensis* via Nrf-2/NF-κB pathway.

7-Deacetylgedunin (**63**, 7-DGD) from *T. sinensis* fruits inhibited inflammation in vitro and in vivo by activating Kelch-like ECH-associated protein-1 (Keap1)/Nrf2/HO-1 signaling in macrophages and LPS-induced septic-shock models [74]. 7-DGD (**63**) also suppressed the proliferation of human synovial fibroblasts from patients with rheumatoid arthritis through the activation of Nrf2/ARE signaling [75]. Deacetylgudunin (**63**, DAG) from *T. sinensis* has excellent anti-inflammatory potential. DAG can inhibit the ASC oligomerization and weaken the interaction of NLR family pyrin domain-containing 3 (NLRP3)-ASC and NLRP3-NEK7 by inhibiting K^+^ efflux and ROS production, which affects assembly of the NLRP3 inflammasome in RAW264.7 cells stimulated by LPS [76]. Toonasinenine A (**99**), B (**101**), C (**102**), D (**103**) and toonafolin (**100**) from TSL EtOH extracts exhibited inhibition of cyclo-oxygenase (COX)-1 and COX-2 and had anti-inflammatory activity [31]. Toonasinemine A (**120**), B (**121**), F (**123**) and I (**73**), which were isolated from dichloromethane (CH_2_Cl_2_) extracts of *T. sinensis* root barks, inhibited NO production significantly at non-toxic concentrations in LPS-activated RAW 264.7 cells [30]. Two new acyclic diterpenes (**141** and **142**) isolated from *T. sinensis* seeds significantly increased the levels of Nrf2/HO-1 and decreased the levels of NF-κB, TNF-*α* and IL-6 in HG-induced GMCs, thereby showing an anti-inflammatory effect [25]. In an acetaminophen (APAP)-treated HepG2 cell model, quercitrin (**180**) from TSL EtOH extracts exhibited anti-inflammatory properties by inhibiting the release of pro-inflammatory mediators, including iNOS and COX-2, as well as the cytokine IL-1β [77]. Polyacetylene compounds isolated from the CH_2_Cl_2_ extracts of *T. sinensis* root barks inhibited NO production in RAW 264.7 cells induced by LPS [46] (Figure 10).

*T. sinensis* has important research value for hypoglycemia. It could be used as medicinal plant material with anti-DM and anti-DN activities. *T. sinensis* extracts and their chemical constituents exert antioxidant and anti-inflammatory effects, mainly by activating the Nrf2/HO-1 pathway and inhibiting the NF-κB pathway in cell and animal models. They have certain curative effects by preventing and relieving oxidative stress and inflammation in DM or DN.

### 5.5. Antitumor Activity

It has been found that TSL aqueous extracts can inhibit the viability of osteosarcoma cell lines (MG-63, Saos-2 and U2OS) by increasing mRNA expression of pro-apoptotic factors. These data suggest that TSL extracts suppress the growth of osteosarcoma cells by inducing apoptosis and are promising anti-osteosarcoma plant extracts [78]. TSL aqueous extracts exhibit anti-leukemia activity in murine mouse blood cells (WEHI-3). After treatment with TSL aqueous extracts, the activities of WEHI-3 cells were reduced significantly, protein expression of cytochrome-C, caspase-3 and Bax increased significantly and protein expression of Bcl-2 decreased significantly. The potential therapeutic effects of TSL aqueous extracts on leukemia were confirmed [79]. TSL aqueous extracts have anti-proliferative effects in human pre-myelocytic leukemia (HL-60) cells by apoptosis induction that is associated with cytochrome-C translocation, caspase-3 activation, poly (ADP-ribose) polymerase (PARP) degradation and dysregulation of Bcl-2 and Bax. Hence, TSL aqueous extracts may have potential as an agent of chemotherapeutic and cytostatic activity in human leukemia [80]. TSL aqueous extracts effectively blocked cell-cycle progression by inhibiting the expression of cyclin D1 and E in lung cancer (A549) cells. In addition, the incubation of these extracts led to the activation of caspase-3-like proteases and apoptotic cell death. These results suggest that *T. sinensis* components have potent anti-cancer effects in vitro. The identification of the useful components in these extracts may lead to the development of a novel class of anti-cancer drugs [81]. The activity of TSL aqueous extracts against small-cell lung cancer is mainly through inhibition of the expression of cyclin D1 and cyclin-dependent kinase 4 (CDK4) in H441 cells (lung adenocarcinoma) and H661 cells (lung large cell carcinoma) (IC_50_ of 0.20 and 0.12 mg/mL, respectively) and the blockade of the cell cycle in the G1 phase. TSL aqueous extracts have an anti-proliferative effect on non-small-cell lung cancer [82,83]. Other studies have shown that treatment with TSL aqueous extracts arrested human renal carcinoma cells in the G0/G1 phase through at decrease in the expression of cyclin D1, CDK2 and CDK4, as well as an induction of the expression of p53 and FOXO3a protein. These results suggest that TSL aqueous extracts may be employed for cancer treatment [84]. TSL aqueous extracts were more cytotoxic than other fractions and exhibited selectivity for ovarian cancer cell lines. TSL aqueous extracts arrested ovarian cancer (SKOV3) cells in the G2/M phase and induced their apoptosis. These results indicate that TSL could be developed into a promising anti-ovarian cancer drug [85]. In addition, TSL aqueous extracts can inhibit the proliferation and induce the apoptosis of hamster cheek pouch squamous cell carcinoma induced by 7,12-dimethylbenz[a]anthracene (DMBA). Downregulation of the protein expression of survivin, X chromosome-linked inhibitor of apoptosis (XIAP), proliferating cell nuclear antigen (PCNA), iNOS and COX-2 and increased apoptotic activity suggested that TSL therapy might aid the prevention of oral cancer [86].

The phenolic in TSL extracts inhibited the proliferation of colon cancer cells, HepG2 cells and breast cancer (MCF-7) cells significantly, with EC_50_ values of 4.00 ± 0.39, 153.16 ± 13.49 and 193.46 ± 14.68 µg/mL, respectively [87]. Gallic acid (**154**) has been identified as the major anti-cancer compound in TSL extracts. It is cytotoxic to prostate cancer (DU145) cells (IC_50_ 15.6 ± 2.1 µg/mL) through ROS generation and mitochondria-mediated apoptosis. These results suggest that gallic acid (**154**) could be developed into a drug to counteract prostate cancer [88]. In addition, gallic acid (**154**) extracted from TSL induced the death of human oral squamous cell carcinoma (HOSCC) cells by upregulating expression of the pro-apoptotic genes TNF-*α*, TP53BP2 and GADD45A and downregulating the expression of the anti-apoptotic genes survivin and cIAP1. There was no effect on normal oral epithelial cells [89]. Betulinic acid (**130**) and 3-oxours-12-en-28-oic acid (**133**) extracted from *T. sinensis* roots inhibited the proliferation of human gastric cancer (MGC-803) cells and human prostate cancer (PC3) cells and led to apoptosis (IC_50_ 17.7 and 13.6 µM, 26.5 and 21.9 µM, respectively) [90]. The limonin-type triterpenoids toonasinenine A (**99**), B (**101**), C (**102**), D (**103**) and toonafolin (**100**) from TSL extracts had significant effects on all tumor cell lines, except glioma cell lines. Toonasinenine I (**114**) and J (**115**) from TSL extracts showed high cytotoxic activity against glioma cell lines [31]. *T. sinensis* extracts and compounds have a wide range of anti-cancer effects. TSL have been studied extensively and could be a source of antitumor drugs. The antitumor activities of *T. sinensis* extracts might be related to their high content of phenolic and limonin-type triterpenoids.

### 5.6. Hepatoprotective Activity

TSL water extracts showed anti-fibrotic effects on rats with liver injury treated with thioacetamide (TAA), including reduced collagen formation and inflammatory factors (TGF-*β*1). These data demonstrate the beneficial effects of TSL water extracts on human liver injury by increasing detoxification and metabolic pathways [90]. Polysaccharides from TSL extracts reduced the levels of alanine aminotransferase (ALT), aspartate aminotransferase (AST) and MDA, increased the activities of SOD, GSH-Px, CAT and GSH, decreased the expression of TNF-*α* and IL-6 and improved the liver injury induced by CCl_4_ in mice. Hence, the polysaccharides in TSL extracts may have a hepatoprotective effect [91]. Polyphenols extracted from the barks and fruits of *T. sinensis* could be used to treat non-alcoholic fatty liver disease by reducing lipoprotein expression in HepG2 cells treated with free fatty acid (FFA), activating the AMPK pathway, promoting lipid metabolism and reducing lipid accumulation [92]. In mice with HFD and alloxan-induced DM, quercetin (**179**) from TSL EtOH extracts alleviated oxidative stress and liver damage significantly according to the measurement of lipid peroxidation, NO content and iNOS activity [57]. Quercitrin (**180**) alleviated APAP-induced liver injury by inhibiting Janus kinase (JNK) and p38 signaling pathways, activating defense genes and inhibiting pro-inflammatory genes in HepG2 cells and animal models [77]. TSL extracts have good hepatoprotective activity and could be used as raw materials to protect against liver damage.

### 5.7. Antiviral and Antibacterial Activity

Extracts from the tender leaves of *T. sinensis* had an obvious inhibitory effect on severe acute respiratory syndrome coronavirus (SARS-CoV), and the selectivity index was 12–17. These leaves may be an important resource for the prevention and control of SARS-CoV [93]. Aqueous extracts of the tender leaves of *T. sinensis* had a highly selective inhibitory effect on the formation of MDCK plaque by the influenza A (H1N1) virus on A549 cells. They inhibited viral attachment by significantly downregulating the expression of adhesion molecules and chemokines (VCAM-1, ICAM-1, E-selectin, IL-8 and fractalkine). These results suggest that aqueous extracts of the tender leaves of *T. sinensis* might be an alternative treatment or prevention for H1N1 virus infection [94].

The essential oil of *T. sinensis* leaves (TSL-EO) contains many sesquiterpenes. Standard broth-microdilution methods were used to evaluate the antibacterial activity of 20 strains of methicillin-sensitive Staphylococcus aureus (MSSA) and methicillin-resistant Staphylococcus aureus (MRSA). TSL-EO therapy revealed inhibitory activity against MSSA and MRSA, and the minimum inhibitory concentration (MIC) was 0.125 and 1 mg/mL, respectively. The biological activity of TSL-EO may be related to the high content of sesquiterpenes [95]. EtOAc extracts of *T. sinensis* shoots contain many polyphenols, glycosides and terpenoids. Antibacterial activity was determined using the agar hole-diffusion method and microdilution method. TSL-EO showed high inhibitory activity against *Staphylococcus aureus*, *Shigella dysentery* and *Escherichia coli* with MIC values of 1.56, 0.78 and 0.39 mg/mL, respectively [50]. In summary, *T. sinensis* has an inhibitory effect on various viruses and bacteria. The antiviral and antibacterial effects of TSL are the most extensive, and they can be used as a potential source of antiviral and antibacterial drugs.

### 5.8. Immunopotentiation

A type of fish (tilapia) that received TSL hot-water extracts (≤8 μg/g) exhibited significant stimulatory effects on non-specific immune mechanisms and disease resistance. TSL hot-water extracts could be used as an immunostimulant in tilapia, but continuous administration may be necessary to maintain the protective response [13]. The immunomodulatory activities of *T. sinensis* seeds were evaluated using cyclophosphamide-induced immunodeficiency in mice. The polysaccharide TSP-3a had a significant immune-restoring activity and enhanced phagocytosis [17]. Rutin (**183**) extracted from TSL methanol extracts could regulate various functions of crustaceans. The survival rate of the littoral shrimp was improved significantly after rutin injection, indicating that a certain dose of rutin could improve the immunity of littoral shrimp to alginolytic *Vibrio* infection [9].

### 5.9. Effects on the Male Reproductive System

TSL aqueous extracts repressed the ROS level, maintained the mitochondrial membrane potential (MMP) and restored sperm motility to improve sperm and testicular function under oxidative stress [96]. Studies have shown that increased levels of oxidative stress may be one of the main causes of decreased semen quality. *T. sinensis* can improve the dynamic activity of human sperm. Primary Leydig cells from mice were purified and tested in vitro. TSL aqueous extracts significantly inhibited the production of testosterone stimulated by basal and human chorionic gonadotropin (HCG) in a dose-dependent manner [97]. The protective effects of TSL EtOH extracts on oxidative stress were studied using H_2_O_2_-treated human sperm. Sperm motility, MMP, denosine triphosphate level and maintenance of chromatin structural integrity were investigated. Therapy with TSL EtOH extracts improved sperm function under oxidative stress by reducing ROS levels and cell death [98]. In conclusion, *T. sinensis* extracts are good natural bioactive products that increase the dynamic activity of human sperm and have great potential for development.

### 5.10. Other Aspects

In addition to the pharmacological effects stated earlier, *T. sinensis* has effects against visceral pain and depression and has neuroprotective effects. The effects of TSL aqueous extracts on antinociceptive activity were studied in a mouse model of visceral pain. The extracts had the same anti-visceral pain properties as those of Rofecoxib and Diclofenac, which have research value in the treatment of refractory visceral pain in humans [99]. Essential oil isolated from *T. ciliata* Roem. var. *yunnanensis* leaves could increase the contents of dopamine (DA), norepinephrine (NE), 5-hydroxytryptamine (5-HT) and brain-derived neurotrophic factor (BDNF) in the hippocampus of rats with chronic mild stress (CMS) and could have anti-depression effects [100]. EtOH extracts of limonin compounds isolated from the young leaves and buds of *T. sinensis* showed significant neuroprotective effects on 6-hydroxydopamine-induced death of human neuroblastoma (SH-SY5Y) cells, with EC_50_ values ranging from 0.27 ± 0.03 to 17.28 ± 0.16 μM in vitro [32]. In summary, the pharmacological activities of different parts of *T. sinensis* are extensive, and it is a natural bioactive product with great potential for development.

Given the current situation of *T. sinensis* resources, the selection, propagation and large-scale cultivation of new varieties should be strengthened. We should also strive to increase the number of populations, improve the quality of varieties and seedlings, as well as perform large-scale and standardized production in suitable growth areas to ensure the sustainable use of resources [101].

## 6. Conclusions

*T. sinensis* is a unique and precious tree species and traditional woody vegetable. It is used widely in the international market and enjoys the reputation of “Chinese mahogany”. It is a famous medicine and edible plant in China, whose leaves, stems, seeds, barks and pericarps can be used as medicines. The chemical constituents and biological activities of *T. sinensis* have been investigated widely.

In this review, 206 compounds were compiled from *T. sinensis*, including triterpenoids, sesquiterpenoids, diterpenoids, sterols, phenols, flavonoids and phenylpropanoids. Terpenoids are the main constituents isolated from plants of the Meliaceae family. With regard to the pharmacological activities described for *T. sinensis*, studies performed using different in vivo and in vitro experimental biological methods have supported most of their traditional medicinal uses. Its extracts and chemical constituents have excellent biological activities, such as anti-DM, anti-DN, antioxidant, anti-inflammatory, antitumor, hepatoprotective, antiviral/antibacterial and immunopotentiation effects.

In summary, the chemical constituents, compound cracking laws and pharmacological activities of different parts of *T. sinensis* were reviewed systematically. This information might highlight the importance of this plant and provide some directions for its future development. In addition, further studies of the biological activities of *T. sinensis* extracts and compounds are needed.

## Figures and Tables

**Table 1:** Chemical compounds isolated from the *T. sinensis* plant.

Comp.	Name	Type	Sources	Ref.
		triterpenoids		
**1**	methyl shoreate	dammarane	stem barks	[23]
**2**	shoreic acid	dammarane	stem barks	[23]
**3**	ocotillone	dammarane	stem barks	[23]
**4**	(20*S*, 24*R*)-epoxydammarane-12, 25-diol-3-one	dammarane	stem barks	[23]
**5**	(20*S*, 24*R*)-epoxydammarane-3*β*, 25-diol-marane-3*β*, 25-diol	dammarane	stem barks	[23]
**6**	richenone	dammarane	stem barks	[23]
**7**	cabralealactone	dammarane	stem barks	[23]
**8**	hollongdione	dammarane	stem barks	[23]
**9**	20-hydroxy-24-dammaren-3-one	dammarane	stem barks	[23]
**10**	(20*S*, 24*S*)-dihydroxydammar-25-en-3-one	dammarane	stem barks	[23]
**11**	cylindrictone D	dammarane	stem barks	[23]
**12**	hispidol B	tirucallane	barks	[24]
**13**	3*β*, 25-dihydroxy-tirucalla-7, 23-diene	tirucallane	seeds	[25]
**14**	3*β*, 23-dihydroxy-tirucalla-7, 24-diene	tirucallane	seeds	[25]
**15**	24, 25-epoxy-3*β*, 23-dihydroxy-7-tirucallene	tirucallane	seeds	[25]
**16**	piscidinol	tirucallane	barks	[24]
**17**	bourjotinolone B	tirucallane	barks	[26]
**18**	(20*S*)-3-oxo-tirucalla-25-nor-7-en-24-oic acid	tirucallane	stem barks	[23]
**19**	4, 4, 14-trimethyl-3-oxo-24-nor-5*α*,13*α*,14*β*,17*α*, 20*S*-chol-7-en-23-oic acid	tirucallane	stem barks	[23]
**20**	(20*S*)-5*α*, 8*α*-epidioxy-3-oxo-24-nor-6,9 (11)-dien-23-oic acid	tirucallane	stem barks	[23]
**21**	Comp. **1** of [24]	*apo*-tirucallane	barks	[24]
**22**	Comp. **2** of [24]	*apo*-tirucallane	barks	[24]
**23**	Comp. **7** of [24]	*apo*-tirucallane	barks	[24]
**24**	Comp. **8** of [24]	*apo*-tirucallane	barks	[24]
**25**	21*α*-*O*-methylmelianodiol	*apo*-tirucallane	pericarps	[1,15,27]
**26**	21*β*-*O*-methylmelianodiol	*apo*-tirucallane	pericarps	[1,15,27]
**27**	Comp. **9** of [24]	*apo*-tirucallane	barks	[24]
**28**	sapelin E acetate	*apo*-tirucallane	barks	[24]
**29**	grandifoliolenone	*apo*-tirucallane	barks	[24]
**30**	bourjotinolone A	*apo*-tirucallane	barks	[24]
**31**	Comp. **4** of [28]	*apo*-tirucallane	seeds, stems	[28]
**32**	Comp. **5** of [28]	*apo*-tirucallane	stems	[28]
**33**	Comp. **6** of [28]	*apo*-tirucallane	stems	[28]
**34**	toonasinensin E	*apo*-tirucallane	seeds, pericarps	[1,15,18,27]
**35**	Comp. **13** of [28]	*apo*-tirucallane	stems	[28]
**36**	Comp. **17** of [28]	*apo*-tirucallane	stems	[28]
**37**	toonasinensin A	*apo*-tirucallane	pericarps	[1,15,27]
**38**	Comp. **6** of [24]	*apo*-tirucallane	barks	[24]
**39**	toonasinensin B	*apo*-tirucallane	pericarps	[1,15,27]
**40**	Comp. **18** of [28]	*apo*-tirucallane	leaves, stems	[28]
**41**	toonasinensin D	*apo*-tirucallane	seeds, pericarps	[1,15,27,28]
**42**	Comp. **22** of [28]	*apo*-tirucallane	leaves	[28]
**43**	toonasinensin C	*apo*-tirucallane	leaves, barks, pericarps	[1,15,24,27,28]
**44**	Comp. **21** of [28]	*apo*-tirucallane	leaves	[28]
**45**	Comp. **1a** of [28]	*apo*-tirucallane	seeds	[28]
**46**	Comp. **7a** of [28]	*apo*-tirucallane	stems	[28]
**47**	Comp. **10a** of [28]	*apo*-tirucallane	leaves	[28]
**48**	Comp. **5** of [24]	*apo*-tirucallane	barks	[24]
**49**	Comp. **3** of [24]	*apo*-tirucallane	barks	[24]
**50**	Comp. **4** of [24]	*apo*-tirucallane	barks	[24]
**51**	Comp. **1** of [28]	*apo*-tirucallane	seeds, leaves, stems	[28]
**52**	Comp. **2** of [28]	*apo*-tirucallane	leaves, stems	[28]
**53**	Comp. **3** of [28]	*apo*-tirucallane	leaves, stems	[28]
**54**	Comp. **7** of [28]	*apo*-tirucallane	leaves, stems	[28]
**55**	Comp. **8** of [28]	*apo*-tirucallane	leaves	[28]
**56**	Comp. **9** of [28]	*apo*-tirucallane	stems	[28]
**57**	Comp. **10** of [28]	*apo*-tirucallane	leaves	[28]
**58**	Comp. **11** of [28]	*apo*-tirucallane	leaves	[28]
**59**	Comp. **12** of [28]	*apo*-tirucallane	leaves	[28]
**60**	Comp. **14** of [28]	*apo*-tirucallane	leaves	[28]
**61**	Comp. **15** of [28]	*apo*-tirucallane	leaves	[28]
**62**	Comp. **16** of [28]	*apo*-tirucallane	leaves	[28]
**63**	7-deacetoxy-7*α*-hydroxygedunin	limonoids	barks	[29]
**64**	gedunin	limonoids	barks	[29]
**65**	7-deacetoxy-7*α*, 11*α-*dihydroxygedunin	limonoids	barks	[29]
**66**	7-deacetoxy-7*α*, 11*β*-dihydroxygedunin	limonoids	barks	[29]
**67**	11*α*-hydroxygedunin	limonoids	barks	[29]
**68**	11*β*-hydroxygedunin	limonoids	barks	[29]
**69**	11-oxogedunin	limonoids	barks	[29]
**70**	11*α*-acetoxygedunin	limonoids	barks	[29
**71**	11*β*-acetoxygedunin	limonoids	barks	[29]
**72**	photogedunin	limonoids	barks	[26]
**73**	toonasinemine I	limonoids	root barks	[30]
**74**	toonasinemine J	limonoids	root barks	[30]
**75**	azadirone	limonoids	barks	[24]
**76**	toonasinemine K	limonoids	root barks	[30]
**77**	toonasinemine L	limonoids	root barks	[30]
**78**	toonasinenine F	limonoids	root barks	[30]
**79**	toonacilianin D	limonoids	leaves	[31]
**80**	toonasinenine H	limonoids	leaves	[31]
**81**	toonasinenine G	limonoids	leaves	[31]
**82**	toonasinenine E	limonoids	leaves	[31]
**83**	toonasinenoids A	limonoids	leaves, buds	[32]
**84**	walsurin D	limonoids	leaves, buds	[32]
**85**	walsurin E	limonoids	leaves, buds	[32]
**86**	toonaciliatone F	limonoids	leaves, buds	[32]
**87**	toonayunnanin B	limonoids	leaves, buds	[32]
**88**	toonasinenoids B	limonoids	leaves, buds	[32]
**89**	6*α*-hydroxyazadiradione	limonoids	leaves, buds	[32]
**90**	trichilenone acetate	limonoids	leaves, buds	[32]
**91**	toonasinenoid E	limonoids	leaves, buds	[32]
**92**	14, 15-epoxynimonol	limonoids	leaves, buds	[32]
**93**	toonasinenoids D	limonoids	leaves, buds	[32]
**94**	toonaciliatone B	limonoids	leaves, buds	[32]
**95**	walsunoid H	limonoids	leaves, buds	[32]
**96**	1*α*-methoxy-12*α*-acetoxydihydrocedrelone	limonoids	leaves, buds	[32]
**97**	dysoxylumosin G	limonoids	leaves, buds	[32]
**98**	toonasinenoids C	limonoids	leaves, buds	[32]
**99**	toonasinenine A	limonoids	leaves	[31]
**100**	toonafolin	limonoids	leaves	[31]
**101**	toonasinenine B	limonoids	leaves	[31]
**102**	toonasinenine C	limonoids	leaves	[31]
**103**	toonasinenine D	limonoids	leaves	[31]
**104**	proceranone	limonoids	root barks	[33]
**105**	6-acetoxyobacunol acetate	limonoids	leaves	[34]
**106**	11*β*-hydroxy-7*α*-obacunyl acetate	limonoids	leaves	[35]
**107**	11-oxo-7*α*-obacunol	limonoids	leaves	[35]
**108**	11-oxo-7*α*-obacunyl acetate	limonoids	leaves	[35]
**109**	7*α*-acetoxydihydronomilin	limonoids	leaves	[34]
**110**	11*β*-hydroxycneorin G	limonoids	leaves	[35]
**111**	toonins A	limonoids	root barks	[33]
**112**	11-oxocneorin G	limonoids	leaves	[35]
**113**	cedrellin	limonoids	leaves	[34]
**114**	toonasinenine I	limonoids	leaves	[31]
**115**	toonasinenine J	limonoids	leaves	[31]
**116**	surenin	limonoids	root barks	[33]
**117**	toonins B	limonoids	root barks	[33]
**118**	carapolide H	limonoids	root barks	[33]
**119**	carapolide I	limonoids	root barks	[33]
**120**	toonasinemine A	limonoids	root barks	[30]
**121**	toonasinemine B	limonoids	root barks	[30]
**122**	toonasinemine C	limonoids	root barks	[30]
**123**	toonasinemine F	limonoids	barks	[26]
**124**	toonasinemine G	limonoids	root barks	[30]
**125**	toonasinemine D	limonoids	barks	[26]
**126**	toonasins B	limonoids	barks	[26]
**127**	toonasinemine E	limonoids	root barks	[30]
**128**	cycloeucalenol	cycloartane	pericarps	[1,15,27]
**129**	24-methylenecycloartanol	cycloartane	pericarps	[1,15,27]
**130**	betulinic acid	other	barks	[26,36]
**131**	betulin	other	barks	[26]
**132**	erythrodiol	other	barks	[26]
**133**	3-oxours-12-en-28-oic acid	other	roots	[36]
**134**	alismoxide	sesquiterpenoids	pericarps	[1,27]
**135**	oplodiol	sesquiterpenoids	pericarps	[1,27]
**136**	gossweilone	diterpenoids	barks	[26]
**137**	phytol	diterpenoids	leaves	[34]
**138**	(9*S*, 10*E*, 16*R*)-9, 16-dihydroxyoctadec-10-ene-12, 14-diyn-1-yl acetate	diterpenoids	barks	[37]
**139**	2, 6, 10, 15-phytatetraene-14-ol	diterpenoids	leaves	[34]
**140**	2, 6, 10-phytatriene-1, 14, 15-triol	diterpenoids	leaves	[34]
**141**	15-tetrahydroxy-3,7, 11, 15, 15-pentamethyl-2, 6, 10-hexadecatriene	diterpenoids	seeds	[25]
**142**	1-*O*-acetyl-12, 14, 15-trihydroxy-3, 7, 11, 15, 15-pentamethyl-2, 6, 10-hexadecatriene	diterpenoids	seeds	[25]
**143**	*β*-sitosterol	sterols	pericarps, barks, roots	[1,27,33,38]
**144**	lawsaritol A	sterols	pericarps	[1,27]
**145**	(3*β*, 7*α*)-7-methoxystigmast-5-en-3-ol	sterols	pericarps	[1,27]
**145**	stigmast-4-ene-3*β*, 6*β*-diol	sterols	pericarps	[1,27]
**147**	5*α*, 8*α*-epidioxy-(22*E*, 24*R*)-ergosta-6, 22-dien-3*β*-ol	sterols	pericarps	[1,27]
**148**	hydroquinone	phenols	pericarps	[1]
**149**	4-hydroxybenzylamine	phenols	pericarps	[1,27]
**150**	protocatechuic acid	phenols	pericarps	[1]
**151**	3, 4-dihydroxybenzoic acid ethyl ester	phenols	pericarps	[1,27]
**152**	3-methoxy-4-hydroxy phenylethanol	phenols	pericarps, roots	[1,27,33]
**153**	coniferyl aldehyde	phenols	pericarps	[1,27]
**154**	gallic acid	phenols	pericarps	[1]
**155**	methyl gallate	phenols	young leaves, pericarps	[1,39]
**156**	ethyl gallate	phenols	young leaves, leaves, stems, fruits, pericarps	[25,34,40,41]
**157**	syringic acid	phenols	roots	[33]
**158**	4-methoxy-6-(2′, 4′-dihydroxy-6′-methylphenyl)-pyran-2-one	phenols	roots	[33]
**159**	aloeemodin	phenols	roots	[33]
**160**	isoscopoletin	phenols	roots	[33]
**161**	trigallic acid	phenols	pericarps	[1]
**162**	7-methoxy trigallic acid	phenols	pericarps	[1]
**163**	5-*O*-galloylquinic acid	phenols	leaves	[12]
**164**	6-*O*-galloyl-D-glucose	phenols	leaves, shoots	[39]
**165**	1, 2, 3-tri-*O*-galloyl-*β*-D-glucopyranose	phenols	leaves, shoots	[39]
**166**	1, 2, 3, 6-tetra-*O*-galloyl-*β*-D-glucopyranose	phenols	leaves, shoots	[39]
**167**	1, 2, 3, 4, 6-penta-*O*-galloyl-*β*-D-glucose	phenols	pericarps, young leaves	[1,39]
		flavonoids		
**168**	(-)-epicatechin	flavan-3-ols	stems	[40]
**169**	(-)-epigallocatechin gallate	flavan-3-ols	leaves	[40]
**170**	(+)-catechin	flavan-3-ols	leaves, woods	[42]
**171**	procyanidin B3	flavan-3-ols	leaves, woods	[42]
**172**	demethoxymatteucinol	flavanones	stems	[40]
**173**	matteucinol	flavanones	stems	[40]
**174**	5, 7-dihydroxy-8-methoxy flavone	flavones	barks	[38]
**175**	kaempferol	flavonols	young leaves	[41]
**176**	kaempferol-3-*O*-*α*-rhamopyranoside	flavonols	young leaves	[41]
**177**	astragalin	flavonols	young leaves	[41]
**178**	kaempferitrin	flavonols	seeds	[43]
**179**	quercetin	flavonols	young leaves	[41]
**180**	quercetin-3-rhamnoside	flavonols	pericarps, young leaves	[1,39]
**181**	quercetin 3-glucoside	flavonols	pericarps	[25]
**182**	quercetin-3-*O*-*α*-L-arabinopyranoside	flavonols	pericarps	[1]
**183**	rutin	flavonols	leaves, shoots	[39]
**184**	myricetin	flavonols	barks	[38]
**185**	myricitrin	flavonols	barks	[38]
**186**	quercetin 3-*O*-(2″-*O*-galloyl)-*β*-D-glucopyranoside	flavonols	leaves	[12]
**187**	cedralins A	phenylpropanoids	leaves	[44]
**188**	toonin C	phenylpropanoids	roots, pericarps	[1,27]
**189**	cedralins B	phenylpropanoids	leaves	[44]
**190**	matairesinol	phenylpropanoids	root barks	[33]
**191**	lyoniresinol	phenylpropanoids	root barks	[33]
**192**	punicatannin C	phenylpropanoids	pericarps	[1,27]
**193**	*α*-tocopherol	others	leaves	[45]
**194**	lutein	others	leaves	[45]
**195**	toonasindiyne A	others	root barks	[46]
**196**	toonasindiyne B	others	root barks	[46]
**197**	toonasindiyne C	others	root barks	[46]
**198**	toonasindiyne D	others	root barks	[46]
**199**	toonasindiyne E	others	root barks	[46]
**200**	toonasindiyne F	others	root barks	[46]
**201**	Comp. 7 of [46]	others	root barks	[46]
**202**	Comp. 8 of [46]	others	root barks	[46]
**203**	Comp. 9 of [46]	others	root barks	[46]
**204**	Comp. 10 of [46]	others	root barks	[46]
**205**	Comp. 11 of [46]	others	root barks	[46]
**206**	Comp. 12 of [46]	others	root barks	[46]

**Table 2:** Pharmacological activities of *T. sinensis*.

Active Constituents	Extraction Solvent	Experimental Model	Regulatory Mechanism ^a^	Ref.
Antidiabetic activity				
leaves extracts	supercritical-CO_2_ fluid	in vivo: STZ induced mice	triglyceride levels↑, adiponectin levels↓	[52]
leaves extracts	water	in vivo: alloxan-induced diabetic Long-Even rats	GLUT4 mRNA (RT-PCR)↑, GLUT4 protein↑	[53]
leaves extracts	50% alcohol/water	in vitro: 3T3-L1 adipocytes treated by calphostin C	cellular glucose uptake↓	[54]
leaves extracts	95% ethanol	in vivo and in vitro	stimulating glucose uptake, ameliorating insulin resistance	[55]
rutin (**183**, leaves)	water	in vivo: insulin-resistant type 2 diabetes mouse model	IRK activity↑, glucose uptake↑	[56]
quercetin (**179**, leaves)	ethyl acetate	in vivo: diabetic mice induced by HFD and alloxan	p65/NF-κB↓, ERK1/2/MAPK↓, caspase-9↓, caspase-3↓	[57]
Antidiabetic nephropathy activity				
seeds extracts	petroleum ether	in vivo: STZ-induced DN rats	TGF-β1↓, Col IV↓, CTGF↓	[58]
seeds extracts	*n*-butanol	in vivo: STZ-induced DN rats	blood glucose↓, urinary albumin↓, kidney index↓, oxidative stress index↓, serum creatinine↓, urea nitrogen levels↓, oxidative stress↓, TGF-β1↓, Col IV↓, CTGF↓	[59]
seeds extracts	*n*-butyl alcohol	in vitro: HG-induced GMCs	ROS↓, p47phox↓, Nrf2↑,NQO1↑, HO-1↑	[60]
seeds extracts	*n*-butyl alcohol	in vivo: STZ-induced DN rats in vitro: HG-induced human renal glomerular endothelial cells	MCP-1↓, ICAM-1↓, p65↓	[61]
kaempferitrin (**178**, seeds)		in vitro: AGEs-induced GMCs	SOD↑, MDA↓, ROS↓, protecte against OS	[43]
kaempferol (**175**, seeds)		in vitro: HG-induced GMCs	ROS↓, MDA↓, SOD↑, TGF-β1↓, Col IV↓, NOX4↓, p22phox↓, Sestrin2↑, AMPK↑	[62]
toonasinensin B (**39**), toonasinensin D (**41**), 21*α*-*O*-methylmelianodiol (**25**), 21*β*-*O*-methylmelianodiol (**26**) (pericarps)		in vitro: HG-induced GMCs	NADPH↓, sorbitol↓	[27]
two acyclic diterpenoids (seeds)		in vitro: HG-induced GMCs	Nrf2/HO-1↑, NF-κB↓, TNF-α↓, IL-6↓	[25]
Antioxidant activity				
leaves, roots, barks extracts	water	in vivo: senescence-accelerated mice in vitro: DPPH·	TBARS↓, SOD↑, CAT↑, GSH-Px↑, DPPH↓ DPPH free-radical activity	[63]
leaves extracts	acetone	in vitro: ORAC, PSC, HepG2 cells, CAA	anti-proliferative effect, antioxidant properties	[64]
leaves extracts, gallic acid (**154**)	water	in vitro: AAPH inducedhuman umbilical vein endothelial cells	ROS↓, MDA↓, SOD/CAT↑, reverse Bax/Bcl-2 dysregulation	[65]
leaves extracts, gallic acid (**154**)	water	in vitro: various oxidative systems, AAPH-induced human erythrocytes	oxidative hemolysis↓, lipid peroxidation↓, SOD↓	[66]
flavonoids, methyl gallate (**155**) (buds)	70% methanol	in vitro: ABTS·+, DPPH·	ABTS and DPPH free-radical activity	[67]
PGG (**167**), EG (**156**) (young leaves)	liquid-liquid refined extraction	in vitro: ABTS·+, DPPH·	ABTS and DPPH free-radical activity	[68]
five flavonols, three derivatives of gallic acid (young leaves)	95% ethanol	in vitro: four chemical-induced oxidative models	significant antioxidant properties	[41]
toonasinenine D (**103**), E (**82**), G (**81**), H (**80**), I (**114**) and J (**115**) (leaves)	95% ethanol	in vitro: ABTS·^+^, DPPH·	strong scavenging activities	[31]
Anti-inflammatory activity				
leaves extracts	water	in vitro: LPS-induced macrophage	HO-1↑, TNF-α↓	[69]
leaves extracts	water	in vitro: RAW264.7 cells treated with LPS	GSH↑, GSH/GSSG↑, reverse the effects of IL-6 and IL-10	[70]
adventitious shoots extracts		in vitro: LPS treated RAW 264.7 cells and propionibacterium acnes-treated HaCaT cells	suppress MAPK pathways	[71]
leaves extracts	water	in vitro: LPS-induced microglial	NO↓, TNF-α↓, iNOS↓	[72]
polyphenols (seeds)	50% acetone	in vivo: a rat model of Parkinson’s disease	p38 MAPK↓, protein levels of infammatory mediators↓	[73]
7-DGD (**63**)		in vivo: LPS-induced septic shock models in vitro: macrophages	activate Keap1/Nrf2/HO-1 signaling	[74]
7-DGD (**63**)		in vitro: human rheumatoid arthritis synovial fibroblast	activate Nrf2/ARE signaling	[75]
DAG (**63**)		in vitro*:* LPS treated RAW 264.7 cells	K^+^ efflux↓, ROS↓	[76]
toonasinenine A (**99**), B (**101**), C (**102**), D (**103**), toonafolin (**100**) (leaves)	ethanol	in vitro	COX-1↓, COX-2↓	[31]
toonasinemine A (**120**), B (**121**), F (**123**), I (**73**) (root barks)	CH_2_Cl_2_	in vitro: LPS-activated RAW 264.7 macrophages	NO↓	[30]
two acyclic diterpenoids (**141**, **142**) (seeds)		in vivo: HG- induced GMCs	Nrf-2/HO-1↑, NF-κB↓, TNF-α↓, IL-6↓	[25]
quercitrin (**180**, leaves)	95% ethanol	in vitro*:* APAP-treated HepG2 cell	iNOS↓, COX-2↓, IL-1β↓	[77]
polyacetylenes	CH_2_Cl_2_	in vitro: LPS treated RAW 264.7 cells	NO↓	[46]
Antitumor activity				
leaves extracts	water	in vitro: osteosarcoma cells	inhibit the activity of MG-63, Saos-2 and U2OS osteosarcoma cells.	[78]
leaves extracts	water	in vitro: WEHI-3 cells	WEHI-3 cells viability↓, cytochrome C↑, caspase-3↑, Bax↑, Bcl-2↓	[79]
leaves extracts	water	in vitro: HL-60 cells	induce cytochrome C translocation, caspase 3 activation, degradation of PARP, dysregulation of Bcl-2 and Bax	[80]
leaves extracts	water	in vitro: A549 lung cancer cells	cyclin D1 and cyclin E↓	[81]
leaves extracts		in vitro: H441 and H661 cells	cyclin D1 and CDK4↓, block the cell cycle in G1 phase, Bcl2↓, Bax↑	[82,83]
leaves extracts	water	in vitro: ccRCC cells	cyclin D1↓, CDK2↓, CDK4↓, p53 ↑, FOXO3a ↑	[84]
leaves extracts		in vitro: ovarian cancer cells	arrest SKOV3 ovarian cancer cells at the G2/M phase	[85]
leaves extracts	water	in vitro: DMBA-induced hamster cheek pouch squamous cells	survivin, XIAP, PCNA, iNOS, and COX-2 proteins↓	[86]
the total phenolic (leaves)	60% ethanol	in vitro: Caco-2, HepG2, MCF-7	inhibit proliferation	[87]
gallic acid (**154**, leaves)		in vitro: DU145 cells	ROS↑, cytotoxic to DU145cells	[88]
gallic acid (**154**, leaves)		in vitro: HOSCC cells	TNF-α↑, TP53BP2↑, GADD45A↑, Survivin↓, cIAP1↓, induces cell death	[89]
betulinic acid (**130**), 3-oxours-12-en-28-oic acid (**133**) (roots)		in vitro: MGC-803 and PC3 cells	inhibite proliferation, led to apoptosis	[36]
toonasinenine A (**99**), B (**101**), C (**102**), D (**103**), toonafolin (**100**) (leaves)	95% ethanol	in vitro: tumor cell lines	significant effects on all tumor cell lines except glioma cell lines	[31]
Hepatoprotective activity				
leaves extracts	water	in vivo: TAA treated liver injury rats	collagen formation↓, TGF-β1↓	[90]
polysaccharide (leaves)	water	in vivo: the liver injury induced by CCl_4_ in mice	ALT↓, AST↓, MDA↓, SOD↑, GSH-Px↑, CAT↑, GSH↑, TNF-α↓, IL-6↓	[91]
polyphenols (barks and fruits)	water	in vitro: FFA-treated HepG2 cells	lipoprotein↓, activating AMPK pathway, lipid metabolism↑, lipid accumulation↓	[92]
quercetin (**179**, leaves)	70% ethanol	in vivo: diabetic mice induced by HFD and alloxouracil	ameliorating oxidative stress in the liver, protects hepatocytes	[57]
quercitrin (**180**, leaves)	95% ethanol	in vivo:APAP-treated HepG2 cell in vitro: APAP-treated animal models	activation of defensive genes and the inhibition of pro-inflammatory genes via the suppressions of JNK and p38 signaling	[77]
Antiviral and antibacterial activity				
tender leaves extracts		in vitro	anti-SARS coronavirus	[93]
tender leaves extracts	water	in vitro	anti-influenza A virus (H1N1)	[94]
sesquiterpene from essential oil (leaves)	*n*-hexane	in vitro	antimicrobial activity against MSSA and MRSA strains	[95]
polyphenols, glycosides, terpenoids contained in shoots extracts	ethyl acetate	in vitro	inhibitory activities against *Staphylococcus aureus*, *Shigella dysenteriae* and *Escherichia coli*	[50]
Immunopotentiation				
leaves extracts	water	in vivo: tilapia	improve the immune response and resistance of tilapia to hydrophilic bacteroides infection	[13]
polysaccharide TSP-3a (seeds)	water	in vivo: CY induced immunodeficiency mice model	significant immune restoring activity and enhance phagocytosis	[17]
rutin (**183**, leaves)	methanol	in vivo	enhance immunity of shrimp	[9]
Effects on the male reproductive system				
leaves extracts	water	in vivo: rats	ROS↓, aintained MMP, restored the sperm motility	[96]
leaves extracts	water	in vitro: primary mouse Leydig cells	inhibited testosterone production	[97]
leaves extracts	ethanol	in vitro: the human spermatozoa treated with H_2_O_2_	ROS↓, cell death↓	[98]
Other aspects				
leaves extracts	water	in vitro: a visceral pain mouse model	anti-visceral pain properties	[99]
essential oil (leaves)	water	in vivo: CMS rats	anti-depression	[100]
limonoids (leaves and buds)	ethanol	in vitro: 6-hydroxydopamine-induced SH-SY5Y cells	neuroprotective effects	[32]

^a^ ↑ upregulation; ↓ downregulation.

## References

[1] Chen Y., Wang F., Ji C.Y., Liu D., Liu X.X., Wang R.S., Li W.Z. (2022). Chemical constituents of the pericarp of *Toona sinensis* and their chemotaxonomic significance. Biochem. Syst. Ecol..

[2] Yang C.C. (2019). Study on Mulching Technology of *Toon sinensis*. Master’s Thesis.

[3] Zhou X.Y. (2005). Studies on the Genus *Toona* of China. Master’s Thesis.

[4] Jiao Z.Y., Zheng J.W., Yan R.C., Zhang Y., Tang L.L. (2019). Summary of main research on multi-functional precious species of *Toon sinensis*. J. Jiangsu For. Sci. Technol..

[5] He B.Z., Yang H.F. (2019). Afforestation technology and benefit analysis of *Toon sinensis*. South China Agric..

[6] Peng W., Liu Y., Hu M., Zhang M., Yang J., Liang F., Huang Q., Wu C. (2019). *Toona sinensis*: A comprehensive review on its traditional usages, phytochemisty, pharmacology and toxicology. Rev. Bras. Farmacogn..

[7] Feng W., Wang M., Cao J., Sun J., Jiang W. (2007). Regeneration of denaturedpolyphenol oxidase in *Toona sinensis* (A. Juss.) Roam. Process. Biochem..

[8] Mu R., Wang X., Liu S., Yuan X., Wang S., Fan Z. (2007). Rapid determination of volatile compounds in *Toona sinensis* (A. Juss.) Roem. by MAE-HS-SPME followed by GC–MS. Chromatographia.

[9] Hsieh T.J., Wang J.C., Hu C.Y., Li C.T., Kuo C.M., Hsieh S.L. (2008). Effects of rutin from *Toona sinensis* on the immune and physiological responses of white shrimp (*Litopenaeus vannamei*) under Vibrio alginolyticus challenge. Fish Shellfish Immun..

[10] Wan X., Lan Z., Yang S., Yang S., Zhu Y., Wang F., Yang W., Chen J. (2022). Investigation of fragmentation pathways of norpimarane diterpenoids by mass spectrometry combined with computational chemistry. Rapid Commun. Mass Spectrom..

[11] Chen H.Y., Lin Y.C., Hsieh C.L. (2007). Evaluation of antioxidant activity of aqueousextract of some selected nutraceutical herbs. Food Chem..

[12] Cheng K.W., Yang R.Y., Tsou S.C., Lo C.S., Ho C.T., Lee T.C., Wang M. (2009). Analysisof antioxidant activity and antioxidant constituents of Chinese toon. J. Funct. Foods.

[13] Wu C.C., Liu C.H., Chang Y.P., Hsieh S.L. (2010). Effects of hot-water extract of *Toona sinensis* on immune response and resistance to Aeromonas hydrophila inoreochromis mossambicus. Fish Shellfish Immun..

[14] Liao J.W., Chung Y.C., Yeh J.Y., Lin Y.C., Lin Y.G., Wu S.M., Chan Y.C. (2007). Safety evaluation of water extracts of *Toona sinensis* Roemor leaf. Food Chem. Toxicol..

[15] Liu D., Wang R.S., Xuan L.L., Wang X.H., Li W.Z. (2020). Two new apotirucallane-type triterpenoids from the pericarp of *Toona sinensis* and their ability to reduce oxidative stress in rat glomerular mesangial cells cultured under high-glucose conditions. Molecules.

[16] Liao J.W., Yeh J.Y., Lin Y.C., Wei M.M., Chung Y.C. (2009). Mutagenicity and safety evaluation of water extract of fermented *Toona sinensis* Roemor leaves. J. Food Sci..

[17] Wang R.S., Zuo M., Ding S.H., Liu B., Meng C., Song B., Li W.Z. (2021). Recovery of immune activity by administration of polysaccharides of *Toona sinensis* and its characterization of major component. Nat. Prod. Res..

[18] Wang M.X., Sheng Z.C., Wu M., Zhang J.L., Chen X.L. (2020). Research progress on chemical constituents and pharmacological effects of leaves of *Toona sinensis*. For. Sci. Technol..

[19] Zhu Y.F., Zhou Q.M., Feng G.B., Song J.B., Tao X.Y. (1999). Antimicrobial test in vitro of cortex toonae and cortex ailanthi. Chin. J. Mod. Appl. Pharm..

[20] Li W.Z., Han W.N., Liu B., Ding S.H., Zhang X.K., Wang R.S. (2017). Extraction of proteins and preliminary characterization of physicochemical properties in *Toona sinensis* fruit. Genet. Mol. Res..

[21] Liu D., Li Y.Q., Li Y.Q., Li W.Z. (2018). Research progress of effective components extraction and bioactivity of *Toona sinensis* seeds. Shandong Chem. Ind..

[22] (2014). China Journal of Traditional Chinese medicine. Toona sinensis recipe. Chin. Med. Distance Educ. China.

[23] Tang J., Xu J., Zhang J., Liu W.Y., Xie N., Chen L., Feng F., Qu W. (2016). Novel tirucallane triterpenoids from the stem bark of *Toona sinensis*. Fitoterapia.

[24] Mitsui K., Saito H., Yamamura R., Fukaya H., Hitotsuyanagi Y., Takeya K. (2007). Apotirucallane and tirucallane triterpenoids from *Cedrela sinensis*. Chem. Pharm. Bull..

[25] Chen Y., Gao H., Liu X.X., Zhou J.Y., Jiang Y.J., Wang F., Wang R.S., Li W.Z. (2022). Terpenoids from the seeds of *Toona sinensis* and their ability to attenuate high glucose-induced oxidative stress and inflammation in rat glomerular mesangial cells. Molecules.

[26] Meng Q.Q., Peng X.R., Lu S.Y., Wan L.S., Wang X., Dong J.R., Chu R., Zhou L., Li X.N., Qiu M.H. (2016). Lactam triterpenoids from the bark of *Toona sinensis*. Nat. Prod. Bioprospecting.

[27] Wang R.S., Liu D., Liu X.X., Liu F., Xuan L.L., Tang Y., Li W.Z. (2022). Cytotoxicity and polyol pathway inhibitory activities of chemical constituents isolated from the pericarp of *Toona sinensis*. Nat. Prod. Res..

[28] Mitsui K., Maejima M., Saito H., Fukaya H., Hitotsuyanagi Y., Takeya K. (2005). Triterpenoids from *Cedrela sinensis*. Tetrahedron.

[29] Mitsui K., Saito H., Yamamura R., Fukaya H., Hitotsuyanagi Y., Takeya K. (2006). Hydroxylated gedunin derivatives from *Cedrela sinensis*. J. Nat. Prod..

[30] Li J.H., Li Y., An F.L., Zhou M.M., Luo J., Jian K.L., Luo J., Kong L.Y. (2016). Limonoids with modified furan rings from root barks of *Toona sinensis*. Tetrahedron.

[31] Hu J., Song Y., Mao X., Wang Z.J., Zhao Q.J. (2016). Limonoids isolated from *Toona sinensis* and their radical scavenging, anti-inflammatory and cytotoxic activities. J. Funct. Foods.

[32] Fu Y.H., Xie Y.T., Guo J.M., Wang X.P., Jiang B., Zhang W., Qiang L., Kong L.Y., Liu Y.P. (2020). Limonoids from the fresh young leaves and buds of *Toona sinensis* and their potential neuroprotective effects. J. Agric. Food Chem..

[33] Dong X.J., Zhu Y.F., Bao G.H., Hu F.L., Qin G.W. (2013). New limonoids and a dihydrobenzofuran norlignan from the roots of *Toona sinensis*. Molecules.

[34] Luo X.D., Wu S.H., Ma Y.B., Wu D.G. (2000). Limonoids and phytol derivatives from *Cedrela sinensis*. Fitoterapia.

[35] Mitsui K., Maejima M., Fukaya H., Hitotsuyanagi Y., Takeya K. (2004). Limonoids from *Cedrela sinensis*. Phytochemistry.

[36] Yang S., Zhao Q., Xiang H., Liu M., Zhang Q., Xue W., Song B., Yang S. (2013). Antiproliferative activity and apoptosis-inducing mechanism of constituents from *Toona sinensis* on human cancer cells. Cancer Cell Int..

[37] Zhou X.L., Wang P.C., Luo Q., Huang X., Chen X., Li J., Liang C.Q. (2017). Study on chemical constituents of bark of Chinese *Toona sinensis*. Chin. Med..

[38] Li G.C., Yu X.X., Liao R.F., Wang D.Y. (2006). Analysis of chemical constituents in bark of *Toona sinensis*. Chin. Hosp. Pharm..

[39] Wang K.J., Yang C.R., Zhang Y.J. (2007). Phenolic antioxidants from Chinese toon (fresh young leaves and shoots of *Toona sinensis*). Food Chem..

[40] Zhao J., Zhou X.W., Chen X.B., Wang Q.X. (2009). *α*-Glucosidase inhibitory constituents from *Toona sinensis*. Chem. Nat. Compd..

[41] Yang H., Gu Q., Gao T., Wang X., Chue P., Wu Q., Jia X. (2014). Flavonols and derivatives of gallic acid from young leaves of *Toona sinensis* (A. Juss.) Roemer and evaluation of their anti-oxidant capacity by chemical methods. Pharmacogn. Mag..

[42] Kakumu A., Ninomiya M., Efdi M., Adfa M., Hayashi M., Tanaka K., Koketsu M. (2014). Phytochemical analysis and antileukemic activity of polyphenolic constituents of *Toona sinensis*. Bioorg. Med. Chem. Lett..

[43] Jiang W.X., Wang R.S., Liu D., Zuo M., Zhao C.Z., Zhang T.L., Li W.Z. (2018). Protective effects of kaempferitrin on advanced glycation end products induce mesangial cell apoptosis and oxidative stress. Int. J. Mol. Sci..

[44] Lee I.S., Kim H.J., Youn U.J., Chen Q.C., Kim J.P., Ha D.T., Ngoc T.M. (2010). Dihydrobenzofuran norlignans from the leaves of *Cedrela sinensis* A. Juss. Helv. Chim. Acta..

[45] Wang S.W., Lee J., Kwon H.S., Lee K.D., Nam S.H., Park K.H., Yang M.S. (2005). Comparison of tyrosinase inhibitory effect of the natural antioxidants from *Cedrela sinensis*. Agric. Chem. Biotechnol..

[46] Xu W.J., Li J.H., Zhou M.M., Luo J., Jian K.L., Tian X.M., Xia Y.Z., Yang L., Luo J., Kong L.Y. (2020). Toonasindiynes A-F, new polyacetylenes from *Toona sinensis* with cytotoxic and anti-inflammatory activities. Fitoterapia.

[47] Li S., Tang J. (2022). Research progress on limonoids in *Xylocarpus* and their biological activities. Chin. Tradit. Herb. Drugs.

[48] Ren W., Xin S.K., Han L.Y., Zuo R., Li Y., Gong M.X., Wei X.L., Zhou Y.Y., He J., Wang H.J. (2015). Comparative metabolism of four limonoids in human liver microsomes using ultra-high-performance liquid chromatography coupled with high-resolution LTQ-Orbitrap mass spectrometry. Rapid Commun. Mass Spectrom..

[49] Suttiarporn P., Chumpolsri W., Mahatheeranont S., Luangkamin S., Teepsawang S., Leardkamolkarn V. (2015). Structures of phytosterols and triterpenoids with potential anti-cancer activity in bran of black non-glutinous rice. Nutrients.

[50] Yang Z., Li L., Chen C.H., Zhang Y.Y., Yang Y., Zhang P., Bao G.H. (2022). Chemical composition and antibacterial activity of 12 medicinal plant ethyl acetate extracts using LC-MS feature-based molecular networking. Phytochem. Anal..

[51] Qian Y.J., Pi W.X., Zhu G.F., Wei W., Lu T.L., Mao C.Q. (2022). Quality evaluation of raw and processed Corni Fructus by UHPLC-QTOF-MS and HPLC coupled with color determination. J. Pharmaceut. Biomed..

[52] Hsieh T.J., Tsai Y.H., Liao M.C., Du Y.C., Lien P.J., Sun C.C., Chang F.R., Wu Y.C. (2012). Anti-diabetic properties of non-polar *Toona sinensis* Roem extract prepared by supercritical-CO_2_ fluid. Food Chem. Toxicol..

[53] Wang P.H., Tsai M.J., Hsu C.Y., Wang C.Y., Hsu H.K., Weng C.F. (2008). *Toona sinensis* Roem (Meliaceae) leaf extract alleviates hyperglycemia via altering adipose glucose transporter 4. Food Chem. Toxicol..

[54] Yang Y.C., Hsu H.K., Hwang J.H., Hong S.J. (2003). Enhancement of glucose uptake in 3T3-L1 adipocytes by *Toona sinensis* leaf extract. Kaohsiung J. Med. Sci..

[55] Liu H.W., Huang W.C., Yu W.J., Chang S.J. (2015). *Toona Sinensis* ameliorates insulin resistance via AMPK and PPARγ pathways. Food Funct..

[56] Hsu C.Y., Shih H.Y., Chia Y.C., Lee C.H., Ashida H., Lai Y.K., Weng C.F. (2014). Rutin potentiates insulin receptor kinase to enhance insulin-dependent glucose transporter 4 translocation. Mol. Nutr. Food Res..

[57] Zhang Y., Dong H., Wang M., Zhang J. (2016). Quercetin isolated from *Toona sinensis* leaves attenuates hyperglycemia and protects hepatocytes in high-carbohydrate/high-fat diet and alloxan induced experimental diabetic mice. J. Diabetes Res..

[58] Li W.Z., Wang X.H., Han W.N., Liu D.M. (2015). Protective effect of petroleum ether fraction of *Toona sinensis* Roem seeds extracts on kidney of diabetic nephropathy rats. Nat. Prod. Res. Dev..

[59] Li W.Z., Wang X.H., Zhang H.X., Mao S.M., Zhao C.Z. (2016). Protective effect of the *n*-butanol *Toona sinensis* seed extract on diabetic nephropathy rat kidneys. Genet. Mol. Res..

[60] Zhong Y.X., Hu S., Wang T.T., Zhang Y.M., Li W.Z., Yu L., Liu Y.Q., Zhang H.X. (2019). *N*-butyl alcohol extract of *Toona sinensis* attenuates the oxidative stress of glomerular endothelial cells induced by high glucose through activating Nrf2. Chin. J. Histochem. Cytochem..

[61] Guo Y.N., Zhong Y.X., Zheng X.R., Yu L., Li W.Z., Zhang H.X. (2019). Extract of *Toona sinensis* improves glomerular endothelial cell inflammation in DN and its potential mechanism. Nat. Prod. Res. Dev..

[62] Xuan L.L., Li Y.Q., Wang H.J., Liu F., Chen Y., Zhao C.Z., Wang R.S., Li W.Z. (2021). Kaempferol inhibited high glucose-induced oxidative stress and extracellular matrix accumulation in glomerular mesangial cells through regulating AMPK/NOX4 pathway. Nat. Prod. Res. Dev..

[63] Liao J.W., Hsu C.K., Wang M.F., Hsu W.M., Chan Y.C. (2006). Beneficial effect of *Toona sinensis* Roemor on improving cognitive performance and brain degeneration in senescence-accelerated mice. Br. J. Nutr..

[64] Shan S.R., Huang X.M., Zhang M.X. (2016). Anti-cancer and antioxidant properties of phenolics isolated from *Toona sinensis* A Juss acetone leaf extract. Trop. J. Pharm. Res..

[65] Yang H.L., Chen S.C., Lin K.Y., Wang M.T., Chen Y.C., Huang H.C., Cho H.J., Wang L., Kumar K.J., Hseu Y.C. (2011). Antioxidant activities of aqueous leaf extracts of *Toona sinensis* on free radical-induced endothelial cell damage. J. Ethnopharmacol..

[66] Hseu Y.C., Chang W.H., Chen C.S., Liao J.W., Huang C.J., Lu F.J., Chia Y.C., Hsu H.K., Wu J.J., Yang H.L. (2008). Antioxidant activities of *Toona Sinensis* leaves extracts using different antioxidant models. Food Chem. Toxicol..

[67] Xu R., Bu Y.G., Zhao M.L., Tao R., Luo J., Li Y. (2020). Studies on antioxidant and α-glucosidase inhibitory constituents of Chinese toon bud (*Toona sinensis*). J. Funct. Foods.

[68] Wang Y.X., Gu D.Y., Liu C., Tang S.S., Wang S., Wang Y., Tang Y. (2023). Enrichment, analysis, identification and mechanism of antioxidant components in *Toona sinensis*. Chin. J. Anal. Chem..

[69] Yang C.J., Chen Y.C., Tsai Y.J., Huang M.S., Wang C.C. (2014). *Toona sinensis* leaf aqueous extract displays activity against sepsis in both in vitro and in vivo models. Kaohsiung J. Med. Sci..

[70] Kuo C.A., Wu S.Y., Lee C.H., Lai Y.R., Lu C.H., Chen P.C., Cheng J.H., Tsai L.Y., Yen K.T., Tsao Y. (2020). *Toona sinensis* modulates autophagy and cytokines in lipopolysaccharide-induced RAW 264.7 macrophages. Biomed. Pharmacother..

[71] Lim H.J., Park I.S., Jie E.Y., Ahn W.S., Kim S.J., Jeong S.I., Yu K.Y., Kim S.W., Jung C.H. (2020). Anti-inflammatory activities of an extract of in vitro grown adventitious shoots of *Toona sinensis* in LPS-treated RAW264.7 and p*ropionibacterium acnes*-treated HaCaT Cells. Plants.

[72] Wang C.C., Tsai Y.J., Hsieh Y.C., Lin R.J., Lin C.L. (2014). The aqueous extract from *Toona sinensis* leaves inhibits microglia-mediated neuroinflammation. Kaohsiung J. Med. Sci..

[73] Zhuang W., Cai M., Li W., Chen C., Wang Y., Lv E., Fu W. (2020). Polyphenols from *Toona sinensiss* seeds alleviate neuroinflammation induced by 6-hydroxydopamine through suppressing p38 MAPK signaling pathway in a rat model of parkinson’s disease. Neurochem. Res..

[74] Chen J.Y., Zhu G.Y., Su X.H., Wang R., Liu J., Liao K., Ren R., Li T., Liu L. (2017). 7-deacetylgedunin suppresses inflammatory responses through activation of Keap1/Nrf2/HO-1 signaling. Oncotarget.

[75] Chen J., Zhu G., Sun Y., Wu Y., Wu B., Zheng W., Ma X., Zheng Y. (2022). 7-deacetyl-gedunin suppresses proliferation of Human rheumatoid arthritis synovial fibroblast through activation of Nrf2/ARE signaling. Int. Immunopharmacol..

[76] Li Q., Tang P., Zhang P., Cui L., Li Y., Li J., Kong L., Luo J. (2022). Inhibition of the P2X7/NLRP3 inflammasome signaling pathway by deacetylgedunin from *Toona sinensis*. J. Nat. Prod..

[77] Truong V.L., Ko S.Y., Jun M., Jeong W.S. (2016). Quercitrin from *Toona sinensis* (Juss.) M. Roem. attenuates acetaminophen-induced acute liver toxicity in HepG2 cells and mice through induction of antioxidant machinery and inhibition of inflammation. Nutrients.

[78] Chen C.H., Li C.J., Tai I.C., Lin X.H., Hsu H.K., Ho M.L. (2017). The fractionated *Toona sinensis* leaf extract induces apoptosis of human osteosarcoma cells and inhibits tumor growth in a murine xenograft model. Integr. Cancer Ther..

[79] Yang H.L., Thiyagarajan V., Liao J.W., Chu Y.L., Chang C.T., Huang P.J., Hsu C.J., Hseu Y.C. (2017). *Toona sinensis* inhibits murine leukemia WEHI-3 Cells and promotes immune response in vivo. Integr. Cancer Ther..

[80] Yang H.L., Chang W.H., Chia Y.C., Huang C.J., Lu F.J., Hsu H.K., Hseu Y.C. (2006). *Toona sinensis* extracts induces apoptosis via reactive oxygen species in human premyelocytic leukemia cells. Food Chem. Toxicol..

[81] Chang H.C., Hung W.C., Huang M.S., Hsu H.K. (2002). Extract from the leaves of *Toona sinensis* roemor exerts potent antiproliferative effect on human lung cancer cells. Am. J. Chin. Med..

[82] Wang C.Y., Lin K.H., Yang C.J., Tsai J.R., Hung J.Y., Wang P.H., Hsu H.K., Huang M.S. (2010). *Toona sinensis* extracts induced cell cycle arrest and apoptosis in the human lung large cell carcinoma. Kaohsiung J. Med. Sci..

[83] Yang C.J., Huang Y.J., Wang C.Y., Wang P.H., Hsu H.K., Tsai M.J., Chen Y.C., Bharath Kumar V., Huang M.S., Weng C.F. (2010). Antiproliferative effect of *Toona sinensis* leaf extract on non-small-cell lung cancer. Transl. Res..

[84] Chen Y.C., Chien L.H., Huang B.M., Chia Y.C., Chiu H.F. (2016). Aqueous extracts of *Toona sinensis* leaves inhibit renal carcinoma cell growth and migration through JAK2/stat3, Akt, MEK/ERK, and mTOR/HIF-2α Pathways. Nutr. Cancer.

[85] Chang H.L., Hsu H.K., Su J.H., Wang P.H., Chung Y.F., Chia Y.C., Tsai L.Y., Wu Y.C., Yuan S.S. (2006). The fractionated *Toona sinensis* leaf extract induces apoptosis of human ovarian cancer cells and inhibits tumor growth in a murine xenograft model. Gynecol. Oncol..

[86] Wang W.C., Chen C.Y., Hsu H.K., Lin L.M., Chen Y.K. (2016). Chemopreventive effect of *Toona sinensis* leaf extract on 7,12-dimethylbenz[a]anthracene-induced hamster buccal pouch squamous cell carcinogenesis. Arch. Oral Biol..

[87] Liu J., You L., Wang C., Liu R. (2012). Antioxidization and antiproliferation of extract from leaves of *Toona sinensis*. Med. Sci..

[88] Chen H.M., Wu Y.C., Chia Y.C., Chang F.R., Hsu H.K., Hsieh Y.C., Chen C.C., Yuan S.S. (2009). Gallic acid, a major component of *Toona sinensis* leaf extracts, contains a ROS-mediated anti-cancer activity in human prostate cancer cells. Cancer Lett..

[89] Chia Y.C., Rajbanshi R., Calhoun C., Chiu R.H. (2010). Anti-neoplastic effects of gallic acid, a major component of *Toona sinensis* leaf extract, on oral squamous carcinoma cells. Molecules.

[90] Fan S., Chen H.N., Wang C.J., Tseng W.C., Hsu H.K., Weng C.F. (2007). *Toona sinensis* Roem (Meliaceae) leaf extract alleviates liver fibrosis via reducing TGFbeta1 and collagen. Food Chem. Toxicol..

[91] Cao J.J., Lv Q.Q., Zhang B., Chen H.Q. (2019). Structural characterization and hepatoprotective activities of polysaccharides from the leaves of *Toona sinensis* (A. Juss) Roem. Carbohydr. Polym..

[92] Chen Y.C., Chen H.J., Huang B.M., Chen Y.C., Chang C.F. (2019). Polyphenol-rich extracts from *Toona sinensis* bark and fruit ameliorate free fatty acid-induced lipogenesis through AMPK and LC3 pathways. J. Clin. Med..

[93] Chen C.J., Michaelis M., Hsu H.K., Tsai C.C., Yang K.D., Wu Y.C., Cinatl J., Doerr H.W. (2008). *Toona sinensis* Roem tender leaf extract inhibits SARS coronavirus replication. J. Ethnopharmacol..

[94] You H.L., Chen C.J., Eng H.L., Liao P.L., Huang S.T. (2013). The effectiveness and mechanism of *Toona sinensis* extract inhibit attachment of pandemic influenza A (H1N1) Virus. Evid.-Based Complement. Altern..

[95] Wu J.G., Peng W., Yi J., Wu Y.B., Chen T.Q., Wong K.H., Wu J.Z. (2014). Chemical composition, antimicrobial activity against Staphylococcus aureus and a pro-apoptotic effect in SGC-7901 of the essential oil from *Toona sinensis* (A. Juss.) Roem. leaves. J. Ethnopharmacol..

[96] Yu B.C., Yu W.J., Huang C.Y., Chen Y.H., Tsai Y.C., Chang C.C., Chang S.J. (2012). *Toona sinensis* leaf aqueous extract improves the functions of sperm and testes via regulating testicular proteins in rats under oxidative stress. Evid.-Based Complement. Altern..

[97] Poon S.L., Leu S.F., Hsu H.K., Liu M.Y., Huang B.M. (2005). Regulatory mechanism of *Toona sinensis* on mouse leydig cell steroidogenesis. Life Sci..

[98] Yu W.J., Yu B.C., Yang F.Y., Chang C.C., Huang C.Y., Chang S.J. (2012). *Toona sinensis* affects reproductive physiology of male. J. Food Drug Anal..

[99] Su Y.F., Yang Y.C., Hsu H.K., Hwang S.L., Lee K.S., Lieu A.S., Chan T.F., Lin C.L. (2015). *Toona sinensis* leaf extract has antinociceptive effect comparable with non-steroidal anti-inflammatory agents in mouse writhing test. BMC Complem. Altern. Med..

[100] Duan D., Chen L., Yang X., Tu Y., Jiao S. (2014). Antidepressant-like effect of essential oil isolated from *Toona ciliata* Roem. var. yunnanensis. J. Nat. Med..

[101] Jiang X. (2009). Research progress of *T. sinensis*. Heilongjiang Sci. Technol. Inf..

